# Mechanosynthesis of Polyureas and Studies of Their Responses to Anions

**DOI:** 10.3390/polym15204160

**Published:** 2023-10-20

**Authors:** Wahab K. A. Al-Ithawi, Rammohan Aluru, Artem V. Baklykov, Albert F. Khasanov, Igor S. Kovalev, Igor L. Nikonov, Dmitry S. Kopchuk, Alexander S. Novikov, Sougata Santra, Grigory V. Zyryanov, Brindaban C. Ranu

**Affiliations:** 1 Chemical Engineering Institute, Ural Federal University, 19 Mira St., Yekaterinburg 620002, Russia; valitkhavi@urfu.ru (W.K.A.A.-I.); rammohan4ever@gmail.com (R.A.); a.f.khasanov@ya.ru (A.F.K.); ekls85@yandex.ru (I.S.K.); rodonid93@mail.ru (I.L.N.); dkopchuk@mail.ru (D.S.K.); sougatasantra85@gmail.com (S.S.); bcranu@gmail.com (B.C.R.); 2 Energy and Renewable Energies Technology Center, University of Technology-Iraq, Baghdad 10066, Iraq; 3 I. Ya. Postovsky Institute of Organic Synthesis of RAS (Ural Division), 22/20 S. Kovalevskoy/Akademicheskaya St., Yekaterinburg 620219, Russia; art.baklykov@gmail.ru; 4 Institute of Chemistry, Saint Petersburg State University, Universitetskaya Nab., 7/9, Saint Petersburg 199034, Russia; a.s.novikov@spbu.ru; 5 Research Institute of Chemistry, Peoples’ Friendship University of Russia (RUDN University), Miklukho-Maklaya Street, 6, Moscow 117198, Russia; 6 School of Chemical Sciences, Indian Association for the Cultivation of Science, Jadavpur, Kolkata 700 032, India

**Keywords:** diaminobiphenyls, ball-milling, polyureas, anions, fluorescence response, fluoride anion detection

## Abstract

Polyureas (PUs) have already found wide practical applications, and various methods of their synthesis have been reported. In this manuscript, we wished to report the very first mechanochemical approach towards aromatic PUs via reactions between isomeric 2,2′-, 3,3′-, and 4,4′-diaminobiphenyls and triphosgene under solvent-free conditions following ball-milling. By using this synthetic approach, both PUs and azomethine-capped Pus were obtained. The fluorescence response of the above-mentioned PUs towards various anions in solutions were studied and selective fluorescence responses towards the hydroxyl and fluoride anions were observed.

## 1. Introduction

In recent decades, mechanosynthesis has become an attractive tool for organic synthesis as a sustainable/greener alternative to conventional solvent-based conditions for carrying out chemical reactions [1,2,3,4]. The most remarkable example of such reactions is solvent-less or solvent-free polymerization for the preparation of functional polymers [5,6], including processes for the construction of coordination polymers [7] or supramolecular polymers [8] under mechanochemical conditions, most commonly under ball-milling ones.

The most common applications of functional polymers include molecular electronics [9,10], photovoltaics [11], energy storage [12], gas storage [13] and/or separation [14], environmental applications [15], and the detection of explosives [16,17], as well as biomedical applications [18]. In this regard, there is a great demand for functional polymers for their application in transport systems [19] and sensory materials for the recognition of biomolecules [20] and bioactive species [21,22].

Among these bioactive species, anions play a key role due to their presence in drugs and their heavy involvement in physiological processes and plant regulation, as well as in the industry; therefore, materials for the detection of anions are of high demand. During the last several decades, significant progress has been made in the field of anion recognition, and a wide variety of synthetic receptors of various geometry and binding sites for anions have been reported [23,24,25]. Among these receptors, urea- and thiourea-bearing molecules can be considered the most attractive ones as they offer directional binding sites for anions due to their ability to bind anions by means of direct hydrogen-bonding interactions under neutral/physiological conditions. Due to the presence of two symmetrical −NH groups, as hydrogen bond donors, on each thiourea moiety these kinds of receptors have already exhibited a wide practical potential for the excellent binding of anions, which mimics the natural binding process in living cells. In the area of supramolecular chemistry, thioureas have been found to be the most efficient ones for the recognition of both single-charged anions, such as halogen anions, including the fluoride anion [26,27], and multi-charged anions of various geometry [28,29,30,31,32], including the amino acid-based anions under physiological pH values [33] and some carboxylate drugs or drug-like molecules [34,35].

It is worth mentioning that polymeric ureas, PUs, have already found many other applications, such as being components of paints and coatings [36], adhesives [37], foams [38], smart hydrogels [39], etc. In addition, as peptidomimetic polymers with high resistance to degradation and low immunogenicity, polyureas have gained increasing interest for bioengineering applications, for instance, as materials for tissue engineering [40] and drug delivery systems [41]. Finally, due to an ability to form hydrogen-bonded architectures, such as self-assembled capsules with tunable guest-binding/encapsulation ability [42], polyureas have already found wide applications as host materials for the separation/capture/release of CO_2_ [43], herbicides [44,45], toxic metal NPs/cations [46,47], and anions [48], as well as other guests [49].

**Figure 1:**
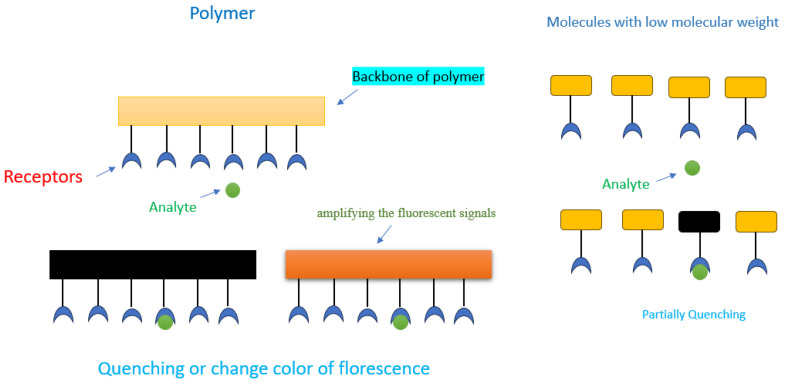
Schematic representation of the difference in sensory signals between polymer- and small molecules-based chemosensors.

Regarding the applications of other polymer-based chemosensors, one can note the following: conjugated polymers have already found wide applications for the detection of various analytes [50,51,52,53,54,55]. According to the commonly accepted concept of the molecular wire [56,57,58], compared to single-molecule-based sensors conjugated polymers provide more binding sites in their backbone, and upon binding of the analyte the whole sensory response of the polymer chain occurs due to the signal amplification (Figure 1). Owing good mechanical properties, polymers can be incorporated into the sensing membrane or sensory elements of the sensing devices [59]. For instance, a conjugated polymer with an incorporated ortho-azonaphthol for ratiometric fluoride ion sensing was recently reported [60]. Due to the deprotonation of the OH group of the azonaphthol moiety in the presence of F^−^ ions, a chromic and fluorogenic component was observed with limits of detection values of 0.96 μM and 2.55 μM, respectively. As another example, a diketopyrrolopyrrole-based conjugated polymer was reported as a chemosensor for the fluoride anion [61]. In the presence of the F^−^ ion, deprotonation occurred and interruption of the electronic energy transfer (EET) took place. In 2008, helical poly(phenylacetylene) with urea moieties appending L-leucine groups was reported [62], and this polymer exhibited a strong colorimetric response to CH_3_CO_2_^−^, as well as other anions, such as spherical F^−^, Cl^−^ Br^−^, I^−^, and multidimensional HSO_4_^−^, NO_3_^−^, and N_3_^−^. These authors also observed distinctive changes in the CD spectra.

In this study, we wished to report the synthesis of aromatic polyureas and the studies on their possible applications for the fluorescence detection of anions, in particular the fluoride anion, which is a known environment pollutant.

## 2. Materials and Methods

Unless otherwise indicated, all common reagents and solvents were used from commercial suppliers without further purification. Silica gel 60 (Kieselgel 60, 230–400 mesh) was used for column chromatography. The diaminobiphenyls **1**–**3** were prepared according to the earlier reported procedures [63,64]. Mechanochemical reactions were performed in the ball mill Retsch PM100, using a 25 mL stainless steel jar with 4 stainless steel 10 mm-diameter milling balls at 500 rpm.

NMR spectra were recorded on a Bruker Avance-400 (or Bruker Avance-500, Bruker, Singapore) spectrometer, 298 K, digital resolution ± 0.01 ppm, using tetramethylsilan (TMS) as the internal standard. ^1^H NMR spectra were recorded at room temperature on a Bruker DRX-400 spectrometer at 400 MHz using DMSO-*d_6_* as a solvent. Hydrogen chemical shifts were referenced to the hydrogen resonance of DMSO-*d_6_* (δ = 2.50 ppm). Gas permeation chromatography (GPC) measurements were performed using an Agilent 1200 chromatograph with an aerosol light scattering detector (ELSD) (Agilent Technologies, Santa Clara, CA, USA) and an Agilent Resipore column, 300 × 7.5 mm—2 pieces in series. UV/Vis absorption spectra were recorded on a Shimadzu UV-1800 spectrophotometer and fluorescence spectra were recorded on a Horiba FluoroMax-4 spectrofluorometer using quartz cells with a 1 cm path length at room temperature.

### 2.1. General Method for the Synthesis of Polyureas ***4***–***6***

Isomeric 2,2(**1**)-, 3,3(**2**)-, or 4,4(**3**)-diaminobiphenyls (100 mg, 0.54 mmol, 1 equiv.), and triphosgene (242 mg, 0.81 mmol, 1.5 equiv.), in the presence of K_2_CO_3_ (540 mg, 3.8 mmol, 7 equiv.), were ball-milled in a 25 mL stainless steel jar with 4 stainless steel 10 mm-diameter milling balls at 500 rpm for 4 h. After that, the resulting mixture was poured in a 10% aqueous solution of HCl, filtrated, and washed with water, EtOH, and acetone.

Polyurea **4**: ^1^H NMR (DMSO-*d*_6_, 400 MHz) δ, ppm: 6.82–7.75 (m, 8H, Ph) and 8.77 (s, 2H, NH-). The yield obtained was 160 mg (77%). Brown powder.

Polyurea **5**: ^1^H NMR (DMSO-*d*_6_, 400 MHz) δ, ppm: 7.12–7.55 (m, 6H, Ph), 7.80 (s, 2H, Ph), and 8.86 (s, 2H, NH-). The yield obtained was 144 mg (70%). Brown powder.

Polyurea **6**: ^1^H NMR (DMSO-*d*_6_, 400 MHz) δ, ppm: 7.51–7.65 (m, 8H, Ph) and 8.97 (s, 2H, NH-). The yield obtained was 198 mg (95%). Off-white powder.

### 2.2. General Method for the Synthesis of Polyureas ***7***–***9***

Isomeric [1,1’-biphenyl]-2,2’-diamine (**1**), [1,1′-biphenyl]-3,3′-diamine, or [1,1′-biphenyl]-4,4′-diamine (benzidine) (100 mg, 0.54 mmol, 1 equiv.), triphosgene (242 mg, 0.81 mmol, 1.5 equiv.), 4-*N,N*-dimethylaminobenzaldehyde (16 mg, 0.08 mmol, 0.15 equiv.), and K_2_CO_3_ (540 mg, 3.8 mmol, 7 equiv.) were ball-milled in a 25 mL stainless steel jar with 4 stainless steel 10 mm-diameter milling balls at 500 rpm for 4 h. After that, the resulting mixture was poured in a 10% aqueous solution of HCl, filtrated, and washed with water, EtOH, and acetone**.**

Polyurea **7**: ^1^H NMR (DMSO-*d_6_*, 400 MHz) δ, ppm: 3.00 (s, 0.06H, CH_3_-ending group), 6.97–7.58 (m, 4H, Ph), and 8.79 (s, 1H, NH-). The yield obtained was 165 mg (80%). Light-yellow powder.

Polyurea **8**: ^1^H NMR (DMSO-*d_6_*, 400 MHz) δ, ppm: 3.00 (s, 0.06H, CH_3_-ending group), 7.00–7.87 (m, 3H, Ph), 7.76 (s, 1H, Ph), and 8.99 (s, 1H, NH-). The yield obtained was 162 mg (78%). Green powder.

Polyurea **9**: ^1^H NMR (DMSO-*d_6_*, 400MHz) δ, ppm: 3.05 (s, 0.06H, CH_3_-ending group), 7.26–7.84 (m, 8H, Ph), and 8.92 (s, 1H, NH-). The yield obtained was 173 mg (83%). Orange powder.

### 2.3. UV/Vis and Fluorescent Titration of the PUs ***4*** and ***7*** with TBAOH and TBAF

UV/Vis and fluorescent titration experiments were carried out with HPLC-grade DMSO as a solvent at room temperature. Solutions of PUs **4** and **7** were prepared by dissolving 3 mg of the samples in 1 mL of DMSO. TBAF was prepared with a concentration of 0.124 M in DMSO; TBAOH was prepared with a concentration of 0.003 M in DMSO. For a typical titration experiment, 10 μL of the solution of a polymer and 1.99 mL of DMSO were placed in a standard quartz cuvette, followed by adding aliquots of the corresponding analyte (10 μL for PU **4** and 20 μL for PU **7**, respectively). The fluorescence emission spectra were measured at room temperature at λ_ex_ = 350 nm.

Stern–Volmer constants were calculated according to Equation (1):

(1) $$\frac{I_{0}}{I}=1+K_{SV}\cdot [Q]$$

where $I_{0}$—fluorescence intensity in the absence of an analyte, *I*—fluorescence intensity in the presence of an analyte, *K_SV_*—Stern–Volmer constant, and [*Q*]—concentration of an analyte.

Limits of detection (LOD) for the F^−^ anion using the probes PU **4** and PU **7** were calculated according to Equation (2) [65]:

(2) $$LOD=\frac{3\times \sigma }{k}$$

where *σ* represents the standard deviation of the chromophore intensity in the absence of an analyte (TBAF) and *k* is the slope of the linear calibration curve.

## 3. Results and Discussion

### 3.1. Mechanosynthesis of the PUs

**Scheme 1:**
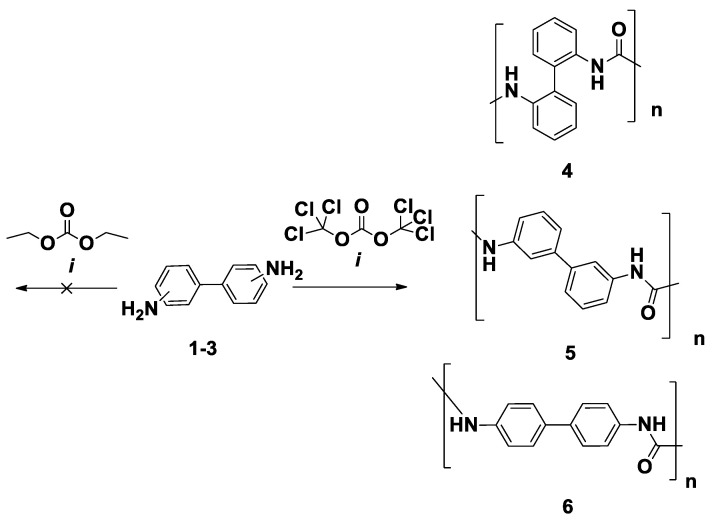
Reagents and conditions: *i*, K_2_CO_3_, 500 rpm, 4 h. Yield up to 95%.

Since the first report of Wöhler [66] on artificial urea preparation, the most common way to prepare thioureas is the reaction between cyanates and amines in solution [67,68,69]. Symmetrical ureas can be obtained from carbamate derivatives via the reaction between amines and carbon dioxide [70], via the industrial, non-catalytic Bazarov’s reaction between ammonia and *sc*CO_2_ [71,72], or via the reaction between amines and phosgene [73,74] or its less-hazardous equivalents [75,76]. As for green methods, in 2016, the green synthesis of polyureas by means of the reaction between CO_2_ and diamines in a media of functional ionic liquid as the catalyst was reported [77]. Among the above-mentioned methods, we selected the one with the utilization of triphosgene for the construction of symmetrical diaryl urea PUs due to its convenience and availability of starting reagents. Moreover, we have recently reported the diaminobiphenyl-based Schiff’s bases as efficient colorimetric probes towards cyanide anions synthesized under mechanochemical conditions [63]. In this work, we applied the mechanochemical conditions for the synthesis of PUs. Thus, PUs **4**–**6** were synthesized by means of the interaction between the equimolar amounts of the corresponding isomeric 4,4- (**1**), 3,3- (**2**), and 2,2- (**3**) diaminobiphenyls and triphosgene in the presence of potassium carbonate under ball-milling at 500 rpm for 4 h (Scheme 1). This reaction seems to be the most attractive for practical application owing its short reaction time (4 h), high yields of the products (up to 95%), no solvent consumption, as well as room temperature conditions. The structures of the PUs **4**–**6** were confirmed using ^1^H NMR and IR- spectroscopy (see ESI for details).

It is important to mention that we have also attempted to carry out a mechanosynthesis of PUs **4**–**6** by means of the reaction between the above-mentioned isomeric diaminobiphenyls and diethyl carbamate as a cheaper and more sustainable alternative to triphosgene [63]. Thus, equimolar amounts of the diaminobiphenyls **1**–**3** and diethyl carbamate were reacted at 500 rpm for 4 to 6 h. However, no PUs of **4**–**6** were obtained and only starting diaminobiphenyls were recovered.

**Scheme 2:**
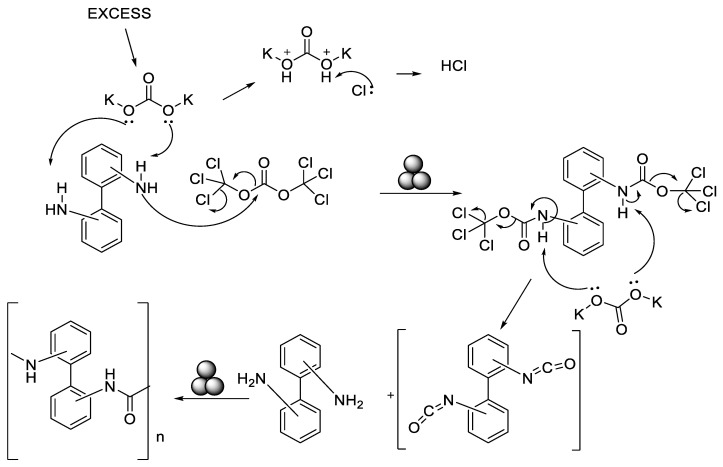
The proposed reaction mechanism for the preparation of PUs **4**–**6**.

The proposed mechanism is presented below (Scheme 2) and involves the nucleophilic attack of the amino group at the carbonyl moiety of triphosgene with the formation of isocyanate and the following addition of the amino group of the second molecule of diaminobiphenyl.

As a next step, molecular weight, as M_n_, measurements were attempted for the obtained PUs **4**–**6** by means of gel permeation chromatography (GPC) under standard conditions (see ESI for details). However, an extremely low solubility of the PUs **4**–**6** in common solvents for GPC (tetrahydrofuran, *o*-dichlorobenzene, trichlorobenzene, and *m*-cresol) was observed, which caused the precipitation of these PUs from the solutions.

**Scheme 3:**
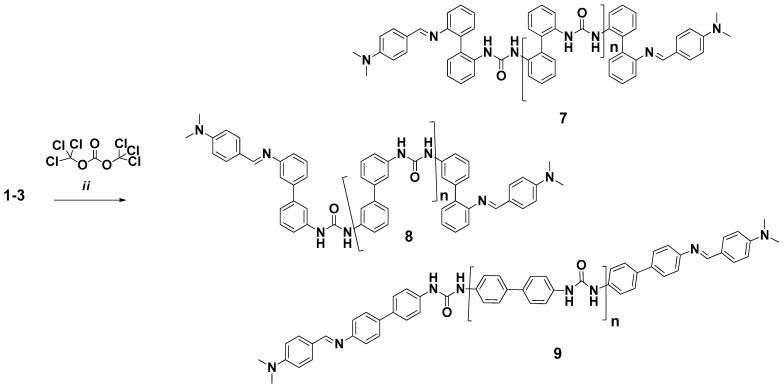
Reagents and conditions: ii, 4-(NMe_2_)C_6_H_4_C(O)H (0.1 equiv.), K_2_CO_3_, 500 rpm, 4 h, and up to 95%.

Another approach for the molecular weight determination was end-group analysis based on ^1^H MMR. For this purpose, the introduction of end groups, such as two *N,N*-dimethylamine moieties, was attempted via the tree-component reaction between the equimolar amounts of the diaminobiphenyls **1**–**3** and triphosgene in the presence of 4-dimethylaminobenzaldehyde (0.1 equiv.) under ball-milling at 500 rpm for 4 h. As a result, the azomethine-capped PUs **7**–**9** were isolated (Scheme 3).

The structures of the PUs **7**–**9** were confirmed using ^1^H and IR- spectroscopy (see ESI for details). Based on the results of the end-group analysis using ^1^H MMR (See ESI for details), the M_n_ values for PUs **7**–**9** were calculated (Table 1). According to ^1^H NMR, the peak of the end group at 3.04 ppm appears near the peak of water in DMSO-d_6_ and in some cases overlaps it. Nevertheless, to overcome this obstacle, the peak of water was removed by means of CF_3_COOD, so that the end group peak became more clearly visible (Figure S31, ESI), and the calculated molecular weight was sufficiently accurate.

Surprisingly, according to the data of Table 1, the highest molecular weight was observed for PU **7**, obtained from the mostly sterically constrained 2,2′-diaminobiphenyl **1**, while the lowest M_n_ was observed for PU **8**, obtained from 3,3′-diaminobiphenyl **2**. Based on the obtained results, one may suggest similar or even higher M_n_ values for the non-capped PUs **4**–**6**.

### 3.2. Photophysical Studies of the PUs and Their Response to Anions

Amino-substituted biphenyl derivatives were reported to exhibit fluorescence properties [78]. Therefore, as a next step, photophysical studies of the PUs **4**–**9** were carried out. Thus, for PUs **4**–**6** in the absorption spectra in the DMSO solution, intensive absorption maxima were observed at 297 nm (**4**), 299 nm (**5**), 327 nm (**6**), 306 nm (**7**), 272 nm (**8**), and 326 nm (**9**). In the fluorescence spectra, emission maxima at 430 nm (**4**), 417 nm (**5**), 372 nm (**6**), 430 nm (**7**), 351 nm (**8**), and 371 nm (**9**) were observed (Figures S16, S17, S28, and S33–S35, ESI).

**Figure 2:**
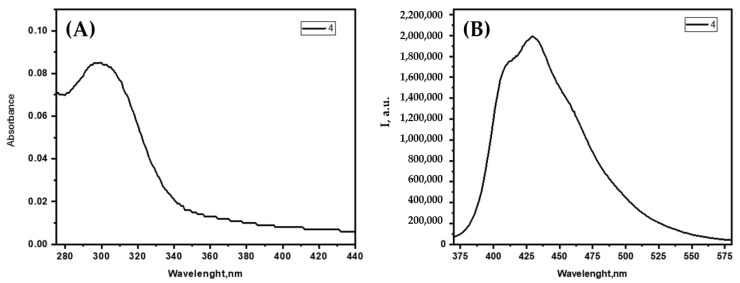
Absorption (**A**) and emission (**B**) spectra of PU **4** in DMSO.

The absorption and emission spectra of PU **4** are shown in Figure 2 as the most representative sample.

According to the literature, thioureas are common receptor units for the recognition of anions of various geometries, and, to the best of our knowledge, PUs have never been reported as chemosensors/probes for the recognition of anions.

**Figure 3:**
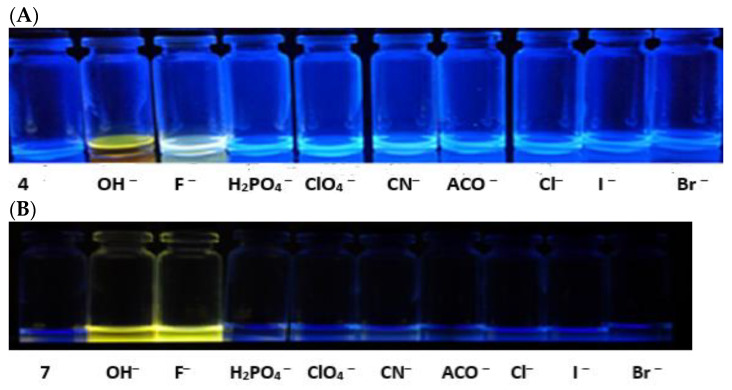
Fluorescence response of PU **4** (**A**) and PU **7** (**B**) towards TBAF in DMSO solution under UV light (λ_ex_ = 365 nm).

**Figure 4:**
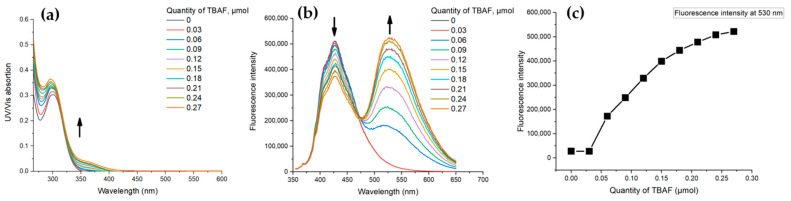
Changes in the absorption (**a**) and emission (**b**) spectra, as well as changes in the emission intensity at 530 nm (**c**) of PU **4** in DMSO in the presence of TBAF.

Therefore, as the next step, the sensory responses of the PUs **4**–**6** in DMSO solutions were recorded in the presence of common anions, such as F^−^, H_2_PO_3_^−^, ClO_4_^−^, CN^−^, Ac^−^, Cl^−^, I^−^, and Br^−^, taken as tetrabutylammonium salts, and the responses of these PUs to the presence of bases (tetrabutylammonium hydroxide, TBAOH) were also studied. Among all anions and PUs **4**–**6**, only in the case of the *ortho*-substituted (2,2′-diaminobiphenyl-based) PU **4** was a well-pronounced fluorescence response observed in the case of the F^−^ anion. Thus, upon the addition of TBAF to the solution of PU **4** in DMSO, a very bright greenish fluorescence was observed in the presence of TBAF (Figure 3A). This response was also observed in the UV and fluorescence spectra (Figure 4).

Thus, upon the addition of increasing amounts of TBAF in DMSO solution in the UV-spectra, a new absorption peak as a shoulder in the region of 350–400 nm was observed (Figure 4a), along with the increase in the intensity of the absorption peak at 297 nm. In the fluorescence spectra taken at the excitation wavelength of 365 nm in DMSO solution, upon the addition of increasing amounts of TBAF, a dramatic quenching of the fluorescence maximum at 430 nm was observed (Figure 4b), along with a bathochromic shift by 100 nm and an appearance of a new emission peak at 530 nm. Calculated limit of detection (LOD) values revealed extremely high values for the PUs in the presence of TBAF—308,630 ppm. This value is several orders of magnitude higher than that reported in the literature (both for small molecules and polymers) [78]. This LOD value is in agreement with the fluorescence titration data, where only a large quantity of TBAF (0.06 μmol) could impact fluorescence (Figure 4c). It should be noted that smaller quantities of TBAF could change the fluorescence of PU **4** under UV excitation at 365 nm to the yellow region (new band at 530 nm) that could be detected by the naked eye. However, due to the polymeric nature of PU **4**, this color change rapidly turned back to the violet region (band at 430 nm).

**Figure 5:**
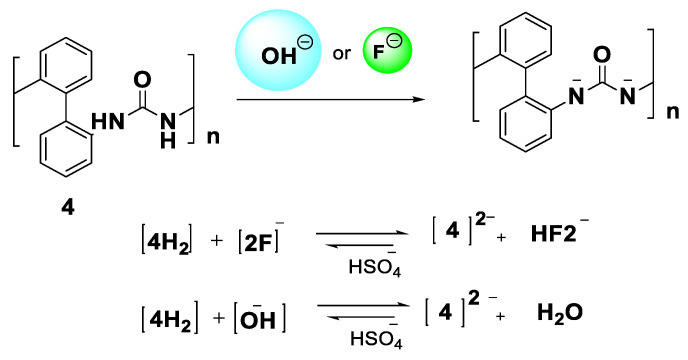
The proposed F^−^/OH^−^ detection mechanism.

In addition, partial quenching of the fluorescence of PU **4** in the presence of OH^−^ was visually observed (Figure 2A). Such behavior of the urea-based sensors in the presence of anions can be explained based on data available in the literature [79]. Thus, in the presence of TBAF and TBAOH, deprotonation of the NH moieties of the urea subunit takes place to result in the formation of the species [4]^2−^, which results in the formation of strong emission bands at 530 nm upon increasing the concentrations of TBAF or TBAOH (Figure 5). The fluorescence arises as a result of the extended delocalized electrons. The addition of a few drops of H_2_SO_4_ results in the disappearance of the strong fluorescence. Deprotonation of PU **4** in the presence of TBAOH and TBAF was confirmed based on ^1^H NMR experiments (Figures S10 and S11, ESI).

In the case of the PUs **5**–**6**, no fluorescence enhancement was observed in the presence of the fluoride anion, as well as other ones (Figures S15–S17, ESI), while a partial quenching was observed in the presence of TBAOH. According to the results of UV titration (Figure S18), upon the addition of increased amounts of TBAOH, a new absorption peak near 350 nm was observed. The presence of TBAOH can be visually detected by the darkening of the colors of the solutions of PU **4**–**6** (for PU **6**, see Figure S22, ESI). Therefore, PUs **4**–**6** can be used as indicators for the determination of OH^−^. It is worth mentioning that in the case of PU **6**, in addition to its fluorescence response to TBAOH, a slight turn-on fluorescence response was observed with the use of TBAAc (Figure S23, ESI).

According to the data of ^1^H NMR, in the case of the PUs **5**–**7** (Figures S10, S12, and S14, ESI), the addition of TBAOH and, in the case of PU **5**, the addition of TBAF (Figure S11, ESI), caused the deprotonation of the urea units [80], and further caused the enhancement in fluorescence due to the stronger delocalization of the electrons [81]. In the case of the other PUs, only a slight fluorescence quenching was observed (Figure S15, ESI). Based on the results of the fluorescence quenching experiments (Figures S16 and S17, ESI), the Stern–Volmer quenching constant of K_SV_ = 12,775 M^−1^ was calculated for PU **5** (Figure S17, ESI).

**Figure 6:**
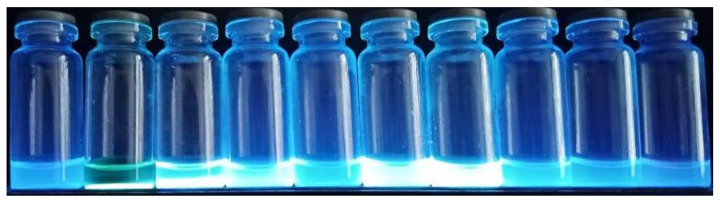
Fluorescence response of PU **6** towards anions in DMSO solution.

Next, the photophysical studies of the azomethine-capped PUs **7**–**9** and their response to anions were studied. In the UV spectra of PUs **7**–**9**, strong absorption bands around 315 nm were observed, and strong emission maxima around 450 nm were detected in the fluorescence spectra (Figure 6). Unlike the PUs **4**–**6**, the azomethine-capped PUs **8**–**9** exhibited almost similar turn-on fluorescence responses to the F ^–^, H_2_PO_4_^–^, CN ^–^, and AcO ^–^ anions. This fact can be explained by the possible reaction between the above-mentioned anions and the azomethine moieties of PUs **8**–**9**, and, in the case of TBAOH, an additional deprotonation of the urea moieties. Similar to the case of the PUs **4**–**6**, a strong fluorescence quenching was observed for the PUs **7**–**9** in the presence of TBAOH (Figure 4). It is worth mentioning that in the case of the PUs **8**–**9**, the addition of TBAF causes a dramatic color change (Figures S24 and S25). Similar to the case of PU **4**, in the case of PU **7**, a fluorescence enhancement was observed in the presence of TBAF (Figure 2B), and a very similar response was observed for TBAOH. The LOD for PU **7** in the presence of TBAF was calculated as 481,698 ppm, which is even higher compared to that obtained with PU **4**.

**Figure 7:**
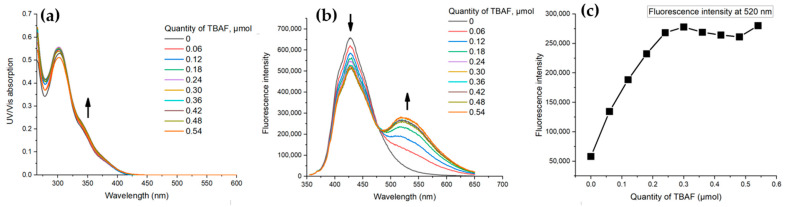
Changes in the absorption (**a**) and emission (**b**) spectra, as well as changes in the emission intensity at 520 nm (**c**) of PU **7** in DMSO and in the presence of TBAF.

Figure 7 represents the UV and fluorescence spectra of PU **7** measured in the presence of an increased concentration of TBAF. Unlike the case of PU **4**, for PU **7**, the addition of increasing amounts of TBAF in DMSO solution in the UV spectra had no influence on the intensity and shape of the absorption peak (Figure 7a). However, in the fluorescence spectra measured at the excitation wavelength of 365 nm in DMSO solution, upon the addition of increasing amounts of TBAF, a quenching of the fluorescence maximum at 430 nm was observed (Figure 7b), along with the bathochromic shift by ca. 90 nm and an appearance of a new emission peak at around 520 nm.

Based on all the above-mentioned results, one can see that among the PUs **4**–**9** only *ortho*-substituted PUs, such as PU **4** and PU **7**, exhibited a selective fluorescence “turn-on” response towards TBAF, while other PUs exhibited no selectivity to F^−^, and all the PUs exhibited a visually detected response to OH^−^. To the best of our knowledge, the herein reported case is the very first example of a selective response to the fluoride anion among the biphenyl-based thioureas [82,83]. The importance of fluoride selective detection is based on its wide presence in dental products, food, drinking water, and tap water [84], and, on the other hand, existing risk health problems caused by the excessive consumption of fluoride, as well as the presence of fluoride in warfare agents [85].

### 3.3. DFT Studies

In order to confirm and explain the selectivity of polymers PU **4**, PU **5**, and PU **6** towards OH^−^ and F^−^ anions, as the next step, DFT calculations were carried out using the ωB97XD/CEP-121G level of theory by means of the Gaussian-09 program. Indeed, the calculated values of ΔG for the model of supramolecular association via hydrogen bonding involving the N–H moieties of PU **4**, PU **5**, and PU **6** with the ACO^−^, Br^−^, Cl^−^, ClO4^−^, CN^−^, F^−^, H_2_PO_4_^−^, I^−^, and OH^−^ anions clearly indicate that supramolecular association processes in the case of F^−^ + PU (**4**–**6**) → PU (**4**–**6**)⋯F^−^ (entries 6, 15, and 24) and OH^−^ + PU (**4**–**6**) → PU (**4**–**6**)⋯OH^−^ (entries 9, 18, and 27) were noticeably higher and, thus, thermodynamically more profitable than the other supramolecular association processes involving the rest of the studied anions (Table 2). These data are in full agreement with the experimentally obtained values.

## 4. Conclusions

In summary, for the first time, a mechanosynthesis of aromatic polyureas as well as azomethine-capped polyureas by means of a condensation reaction between isomeric diaminobiphenyls and triphosgene under ball-milling conditions in the absence of a solvent was reported. For the azomethine-capped polyureas, their molecular weight of 7.4–15.4 KDa was calculated by means of ^1^H NMR end-group analysis. For the first time, the fluorescence properties and fluorescence response of these PUs to anions in solution was studied. All the PUs exhibited blue fluorescence with a maxima of around 450 nm in DMSO solutions. For all the PUs, a dramatic fluorescence response to the OH^−^ anion was observed, and for the 2,2′-diaminobiphenyl-based PUs a selective fluorescence response to the F^−^ anion was observed, yet with reasonably high LOD values of 308,630 ppm and 481,698 ppm for PU **4** and PU **7**, respectively. Based on the obtained results, the herein reported PUs can be considered as colorimetric and fluorescence-based probes for the visual detection of the OH^−^ anion, and 2,2′-diaminobiphenyl-based PUs can be considered as fluorescence “turn-on” probes for the fluoride anion in solution, while other PUs exhibited either low or no selectivity to such an anion as well as other anions.

## Figures and Tables

**Table 1:** Results of the end-group analysis of the PUs **7**–**9**.

PUs	7	8	9
M_n_, Da	15,448	7453	12,192

**Table 2:** Calculated values of ΔG for the model of supramolecular association processes via hydrogen bonding involving the N–H moieties of PU **4**, PU **5**, and PU **6** with the ACO^−^, Br^−^, Cl^−^, ClO_4_^−^, CN^−^, F^−^, H_2_PO_4_^−^, I^−^, and OH^−^ anions.

Entry	Model Supramolecular Association Process	ΔG, kcal/mol
PU **4**		
1	ACO^−^ + PU4 → PU4⋯AcO^−^	−29
2	Br^−^ + PU4 → PU4⋯Br^−^	−19
3	Cl^−^ + PU4 → PU4⋯Cl^−^	−25
4	ClO_4_^−^ + PU4 → PU4⋯ClO_4_^−^	−10
5	CN^−^ + PU4 → PU4⋯CN^−^	−25
6	F^−^ + PU4 → PU4⋯F^−^	−56
7	H_2_PO_4_^−^ + PU4 → PU4⋯H_2_PO_4_^−^	−21
8	I^−^ + PU4 → PU4⋯I^−^	−15
9	OH^−^ + PU4 → PU4⋯OH^−^	−61
PU **5**		
10	ACO^−^ + PU5 → PU5⋯AcO^−^	−35
11	Br^−^ + PU5 → PU5⋯Br^−^	−25
12	Cl^−^ + PU5 → PU5⋯Cl^−^	−31
13	ClO_4_^−^ + PU5 → PU5⋯ClO_4_^−^	−13
14	CN^−^ + PU5 → PU5⋯CN^−^	−31
15	F^−^ + PU5 → PU5⋯F^−^	−61
16	H_2_PO_4_^−^ + PU5 → PU5⋯H_2_PO_4_^−^	−25
17	I^−^ + PU5 → PU5⋯I^−^	−21
18	OH^−^ + PU5 → PU5⋯OH^−^	−67
PU **6**		
19	ACO^−^ + PU6 → PU6⋯AcO^−^	−36
20	Br^−^ + PU6 → PU6⋯Br^−^	−25
21	Cl^−^ + PU6 → PU6⋯Cl^−^	−31
22	ClO_4_^−^ + PU6 → PU6⋯ClO_4_^−^	−14
23	CN^−^ + PU6 → PU6⋯CN^−^	−32
24	F^−^ + PU6 → PU6⋯F^−^	−61
25	H_2_PO_4_^−^ + PU6 → PU6⋯H_2_PO_4_^−^	−28
26	I^−^ + PU6 → PU6⋯I^−^	−20
27	OH^−^ + PU6 → PU6⋯OH^−^	−67

## Data Availability

The data presented in this study are available on request from the corresponding author.

## Supplementary Materials

The following supporting information can be downloaded at: https://www.mdpi.com/article/10.3390/polym15204160/s1, Figures S1–S6: 1H NMR of PU 4–9; Figures S7–S9: IR spectra of PU 4–6; Figures S10–S14: 1H NMR of PU 4,5,9 with addition of TBAF or TBAOH; Table S1: Photophysical properties of the PUs 4, 5, 6 and their complexes with F (-); Figure S15–S28: UV/Vis, colorimetric and fluorescence responses of PU 4-under addition of analytes; Figure S29: 1H NMR of PU 7 in DMSO-d6; Figure S30: IR-spectra of PU 7; Figure S31: 1H NMR spectra of PU 8 in DMSO-d6; Figure S32: IR spectra of PU 8; Figure S33: Absorption and emission spectra of PU 7 in DMSO; Figures S34 and S35: UV/Vis responses of PU 7–9 in the presence of TBAF; Figure S36: Mass spectrum of 1,1′-([1,1′-biphenyl]-2,2′-diyl)bis(3-phenylurea) and1-(2′-amino-[1,1′-biphenyl]-2-yl)-3-phenylurea; Table S2: Results of intraday and interday experiments on synthesis of PUs; Figure S37: Change of FL emission intensity of PU 4 at 530 nm with different aliquot of TBAF; Table S3: Data for Figure S37; Tables S4: Calculated Gibbs free energies for optimized equilibrium model structures. Reference [86] are cited in the supplementary materials.
