# Supplementary material for: Mechanosynthesis of Polyureas and Studies of Their Responses to Anions
Source: Polymers (Basel). 2023 Oct 20;15(20):4160. doi: 10.3390/polym15204160 (PMC10611254; doi:10.3390/polym15204160)
Supplement: Supplementary file 1 [file polymers-15-04160-s001.zip › polymers-2508038-supplementary.pdf]

Supplementary file

Mechanosynthesis of polyureas and studies of their response to anions

Wahab K. A. Al-Ithawi[1,2], Rammohan Aluru[1], Artem V. Baklykov[3], Albert F. Khasanov[1], Igor S. Kovalev[1], Igor L. Nikonov[1,3], Dmitry S. Kopchuk [1,3], Alexander S. Novikov[4,5], Sougata Santra[1], Grigory V. Zyryanov[1,3*] and Brindaban C. Ranu[1,6]

[1] Chemical Engineering Institute, Ural Federal University, 19 Mira St., Yekaterinburg 620002, Russia; valitkhavi@urfu.ru (W.K.A.A.-I.); rammohan4ever@gmail.com (R.A.); art.baklykov@gmail.ru (A.V.B.); a.f.khasanov@ya.ru (A.F.K.); ekls85@yandex.ru (I.S.K.); rodonid93@mail.ru (I.L.N.); dkopchuk@mail.ru (D.S.K.); sougatasantra85@gmail.com (S.S.); bcranu@gmail.com (B.C.R.)

[2] Energy and Renewable Energies Technology Center, University of Technology-Iraq, Baghdad 10066, Iraq

[3] I. Ya. Postovsky Institute of Organic Synthesis of RAS (Ural Division), 22/20 S. Kovalevskoy/Akademicheskaya St., Yekaterinburg 620219, Russia

[4] Institute of Chemistry, Saint Petersburg State University, Universitetskaya Nab., 7/9, Saint Petersburg 199034, Russia; a.s.novikov@spbu.ru (A.S.N.)

[5] Research Institute of Chemistry, Peoples' Friendship University of Russia (RUDN University), Miklukho-Maklaya Street, 6, Moscow 117198, Russia; novikov-as@rudn.ru (A.S.N.)

[6] School of Chemical Sciences, Indian Association for the Cultivation of Science, Jadavpur, Kolkata 700 032, India; bcranu@gmail.com (B.C.R.)

* Correspondence: gvzyryanov@gmail.com; Tel.: +73433754501

Experimental part

General method for the synthesis of Polyureas PUs.

For the synthesis of polyurea, isomeric 4,4-(1), 3,3(2) and 2,2(3) -diaminobiphenyls with 1.5 eq of triphosgene in 7 eq. Potassium carbonate as catalyst with 4 balls for 4 hours at a speed rotation of 500 rpm. after the reaction completed was washed by 10% aqueous solution of HCl, water, EtOH and acetone.

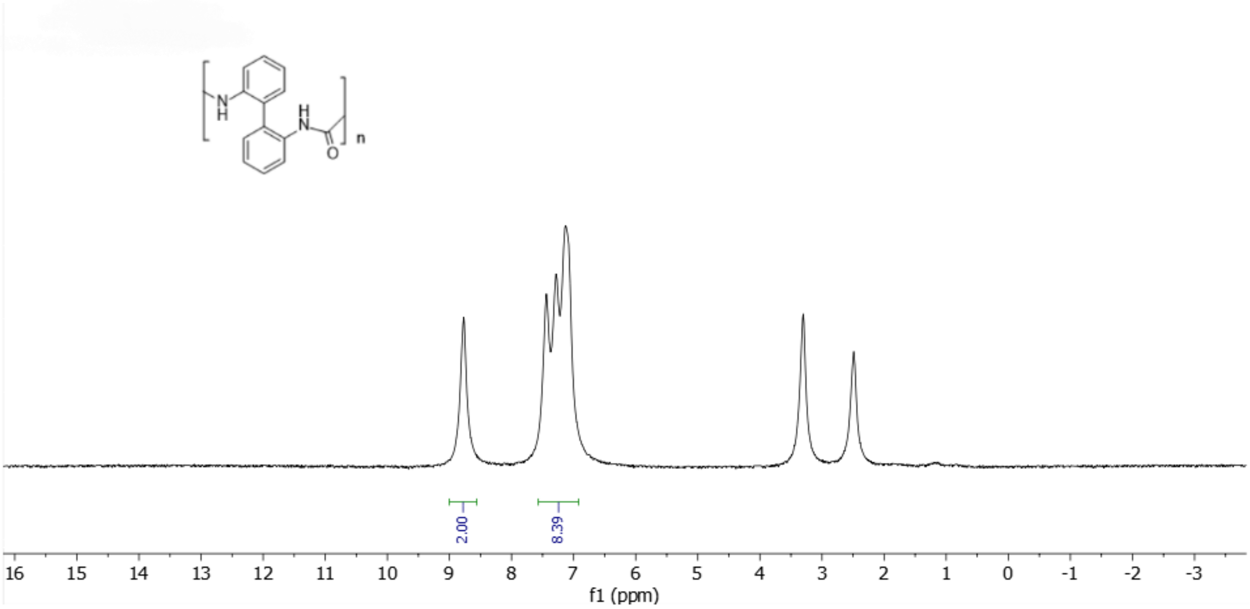

Figure S1. ^{1}H NMR spectrum of the (4) in DMSO-d_6 at r.t

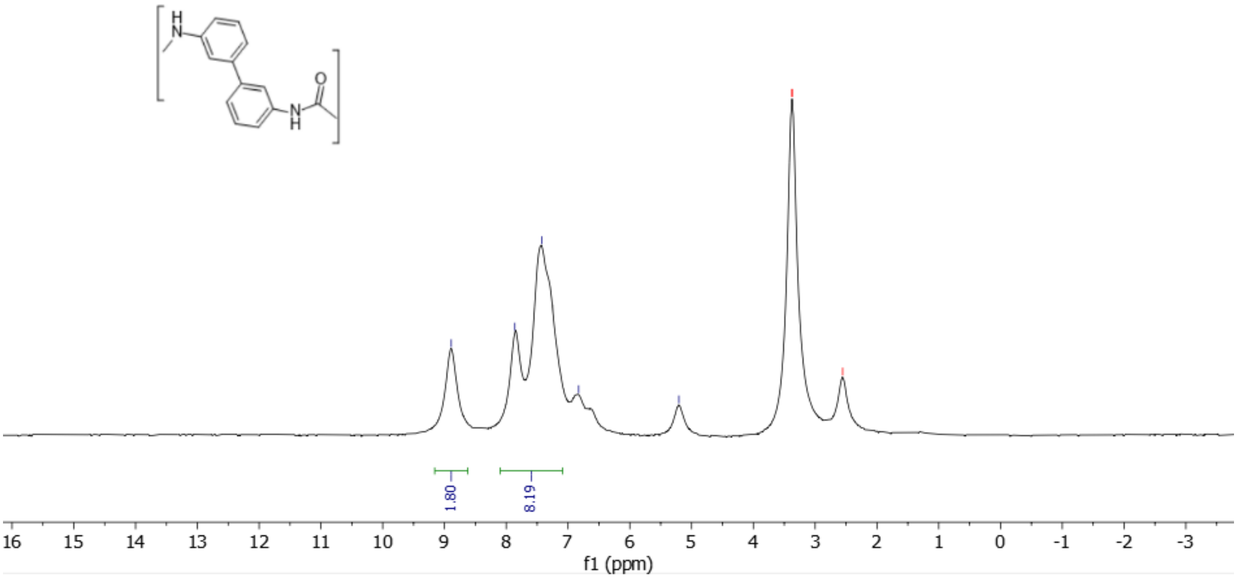

Figure S2. ^{1}H NMR spectrum of the (5) in DMSO-d_6 at r.t

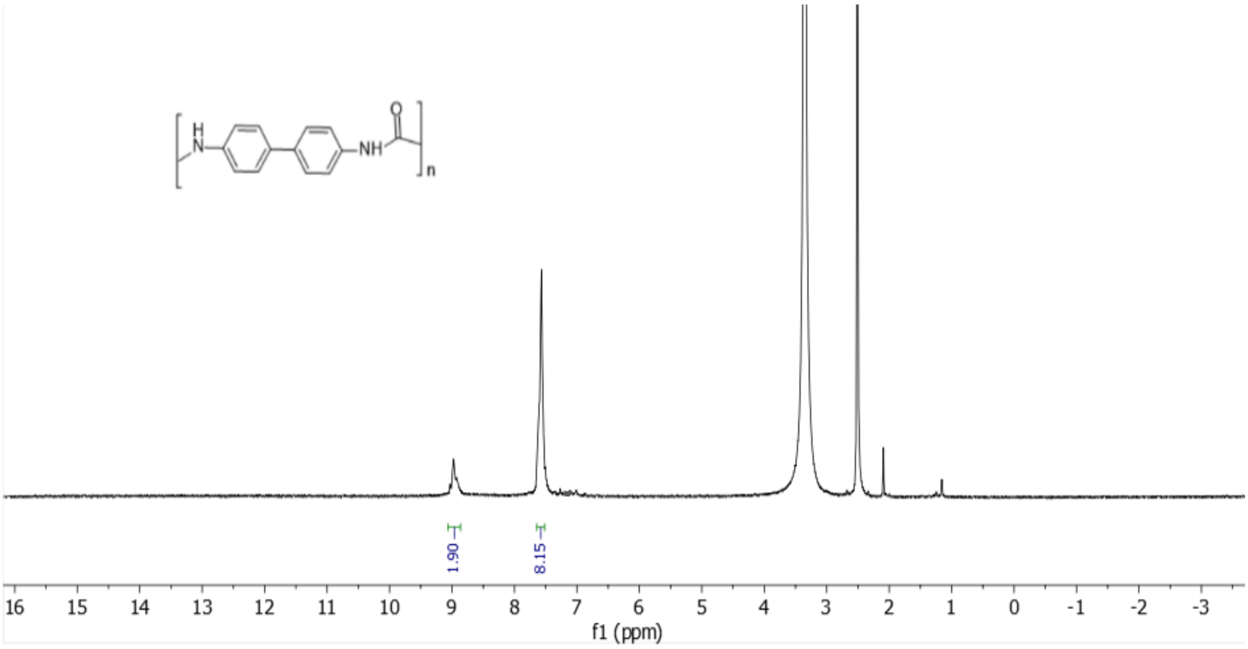

Figure S3. 1H NMR spectrum of the (6) in DMSO-d_6 at r.t

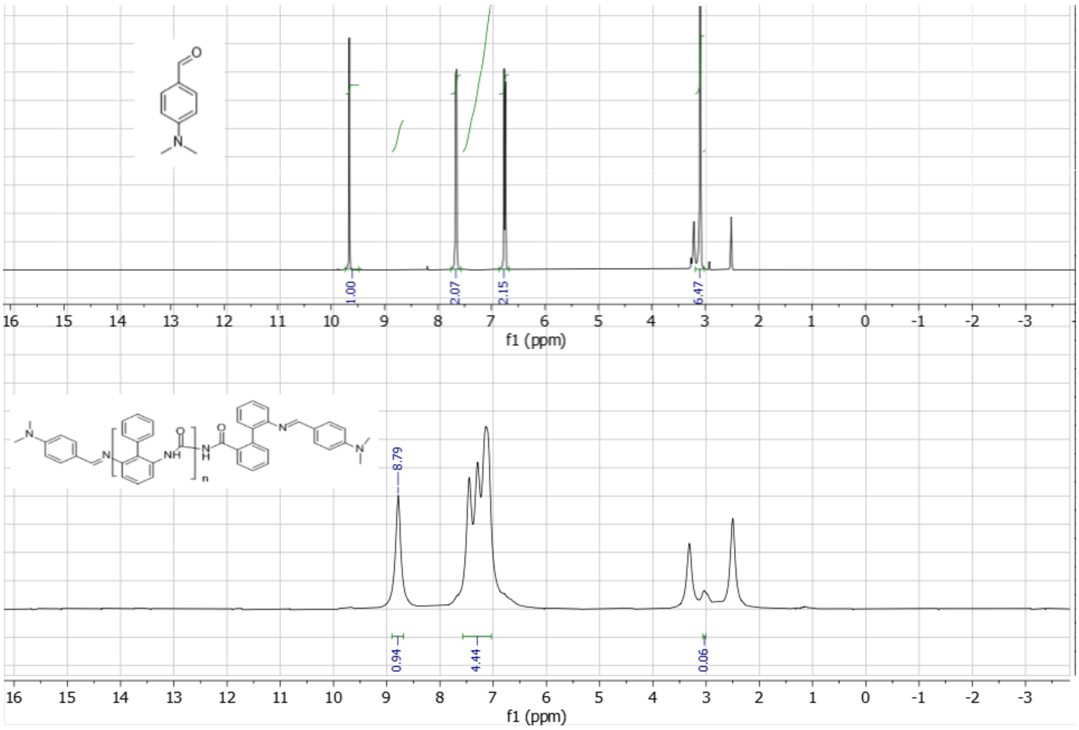

Figure S4. ^{1}H NMR spectrum of the (7) in DMSO-d_6 at r.t with N,N-dimethylamine moiety for determination molecular weight (Mn) of PUs .

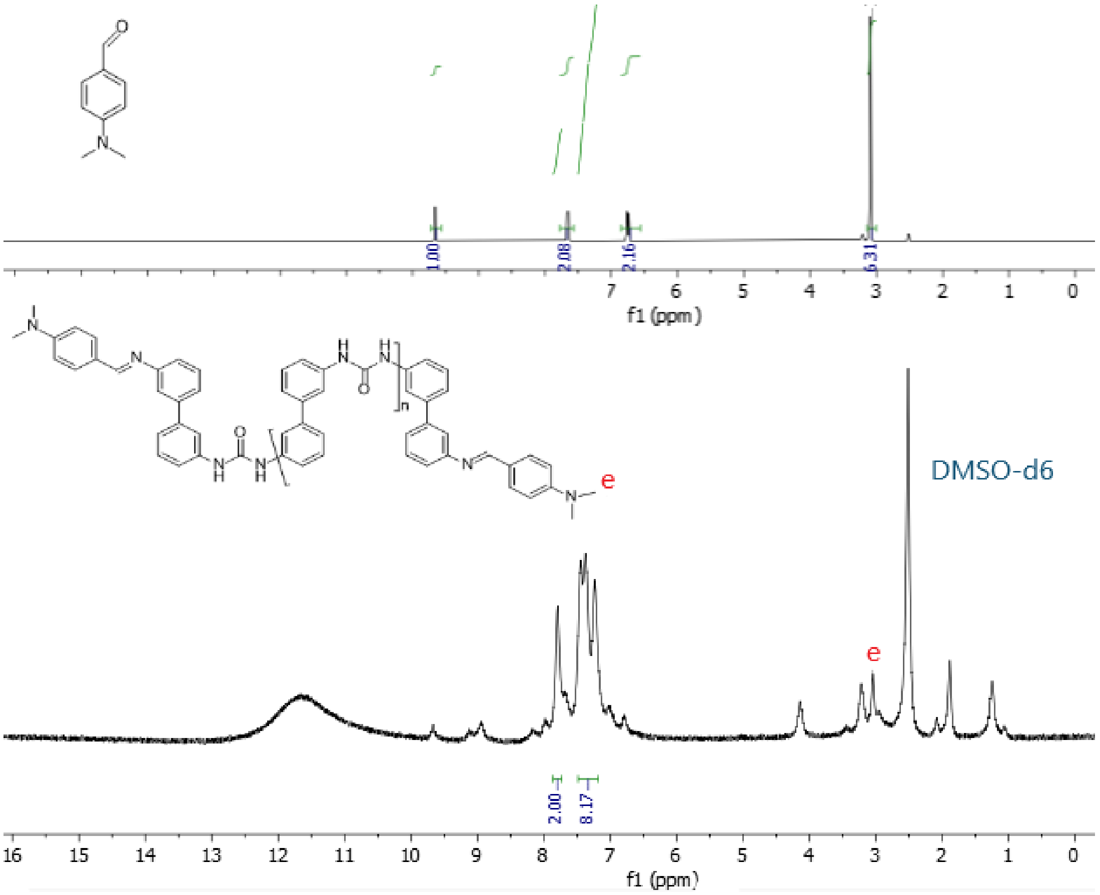

Figure S5. ^{1}**H NMR spectrum of the (8) in DMSO-d6 at r.t with N, N-dimethylamine moiety for determination molecular weight (Mn) of PUs .**

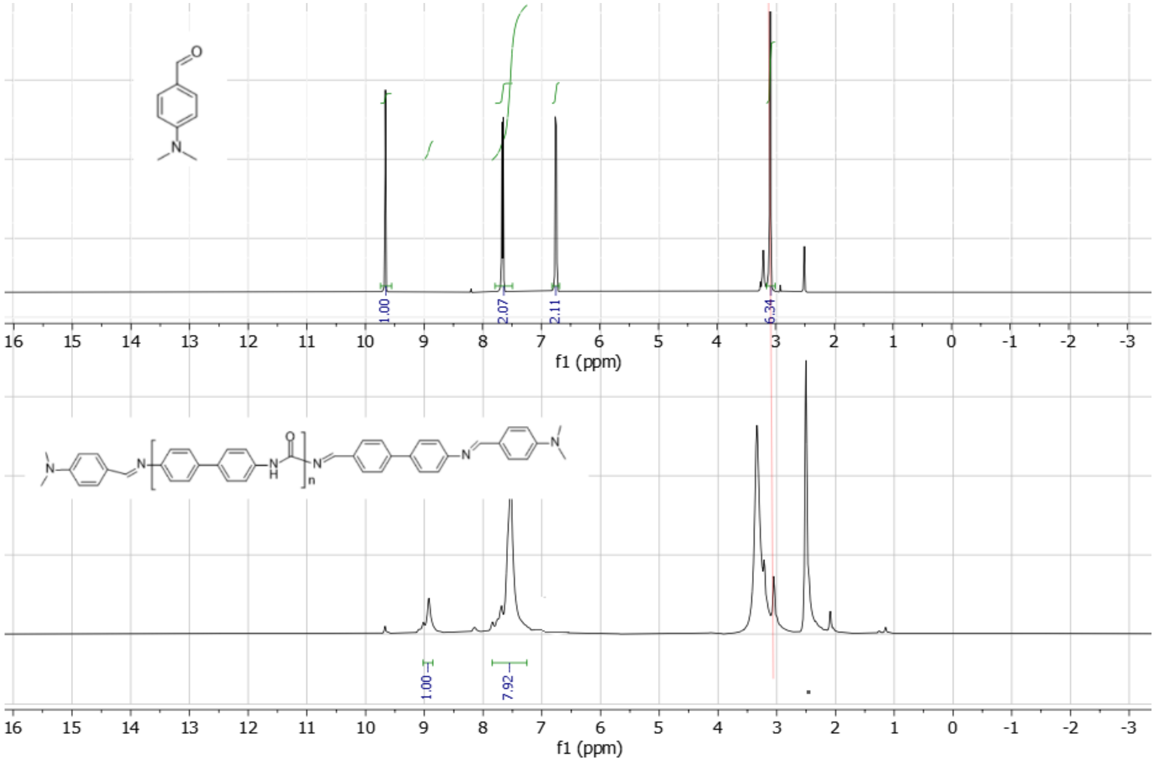

Figure S6. ^{1}**H NMR spectrum of the (9) in DMSO-*d*$_6$ at r.t with N, N-dimethylamine moiety for determination molecular weight (Mn) of PUs.**

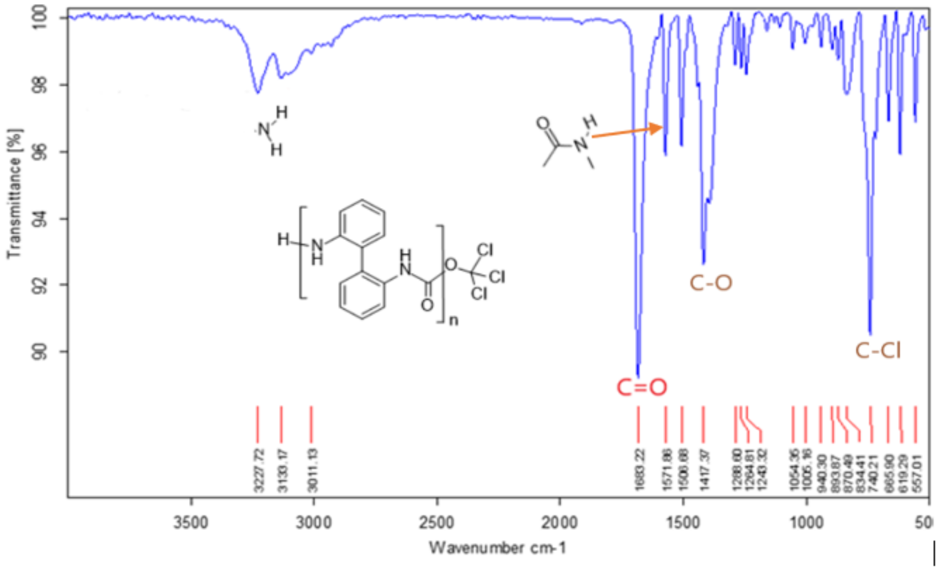

Figure S7 IR spectrum of PU (4)

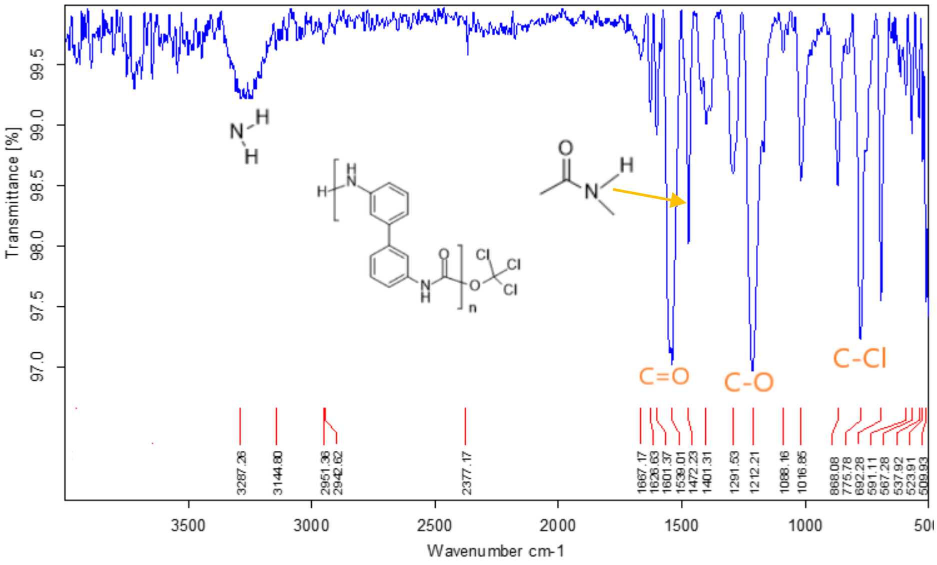

Figure S8 IR spectrum of PU (5)

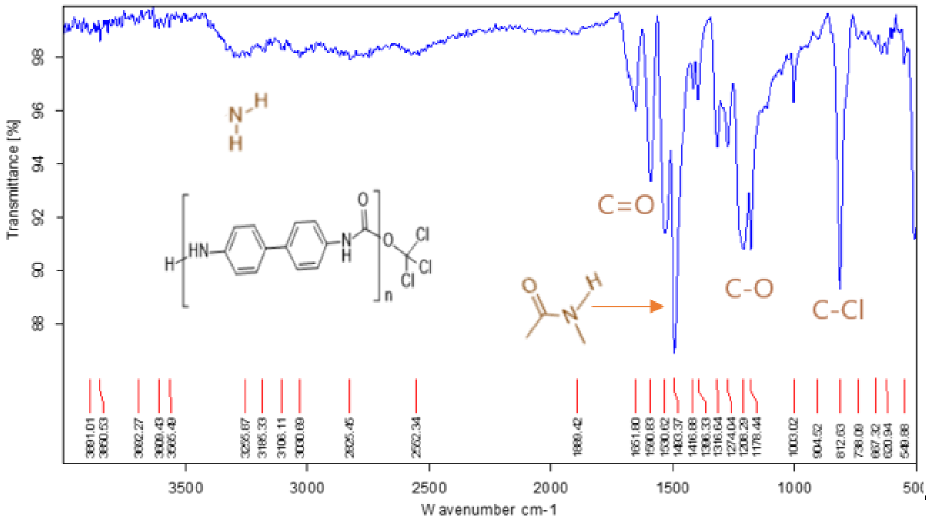

Figure S9 IR spectrum of PU (6)

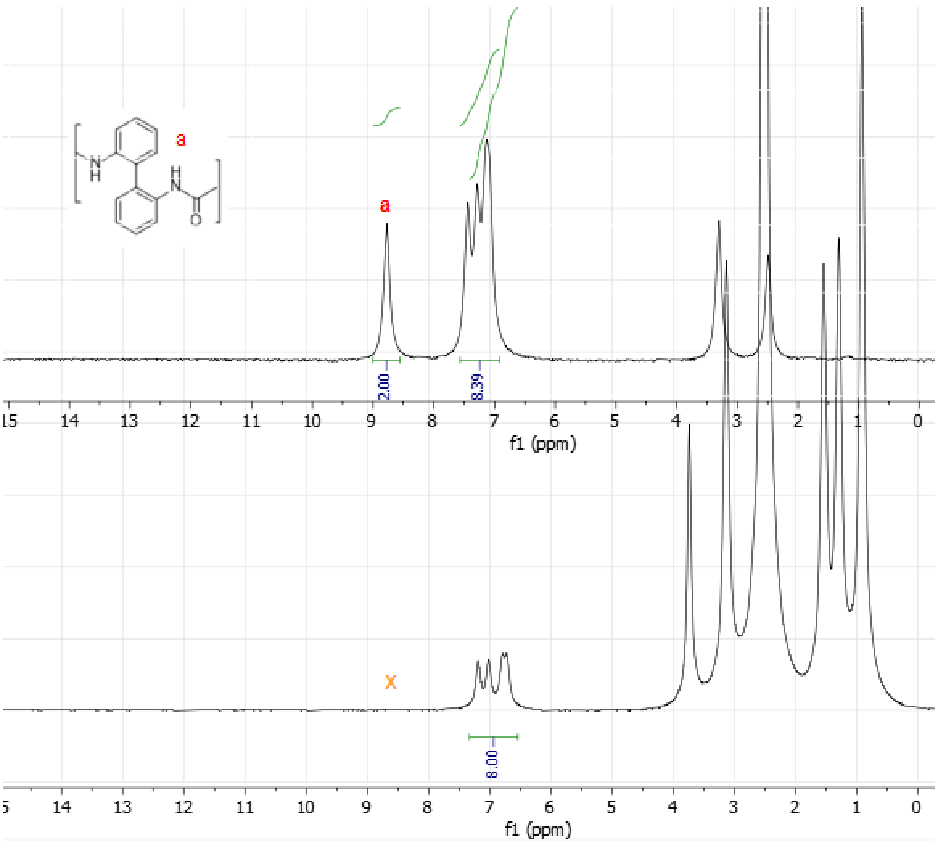

Figure S10. ^{1}H NMR spectrum of the (4) in DMSO-d_6 at r.t. with additional TBAOH

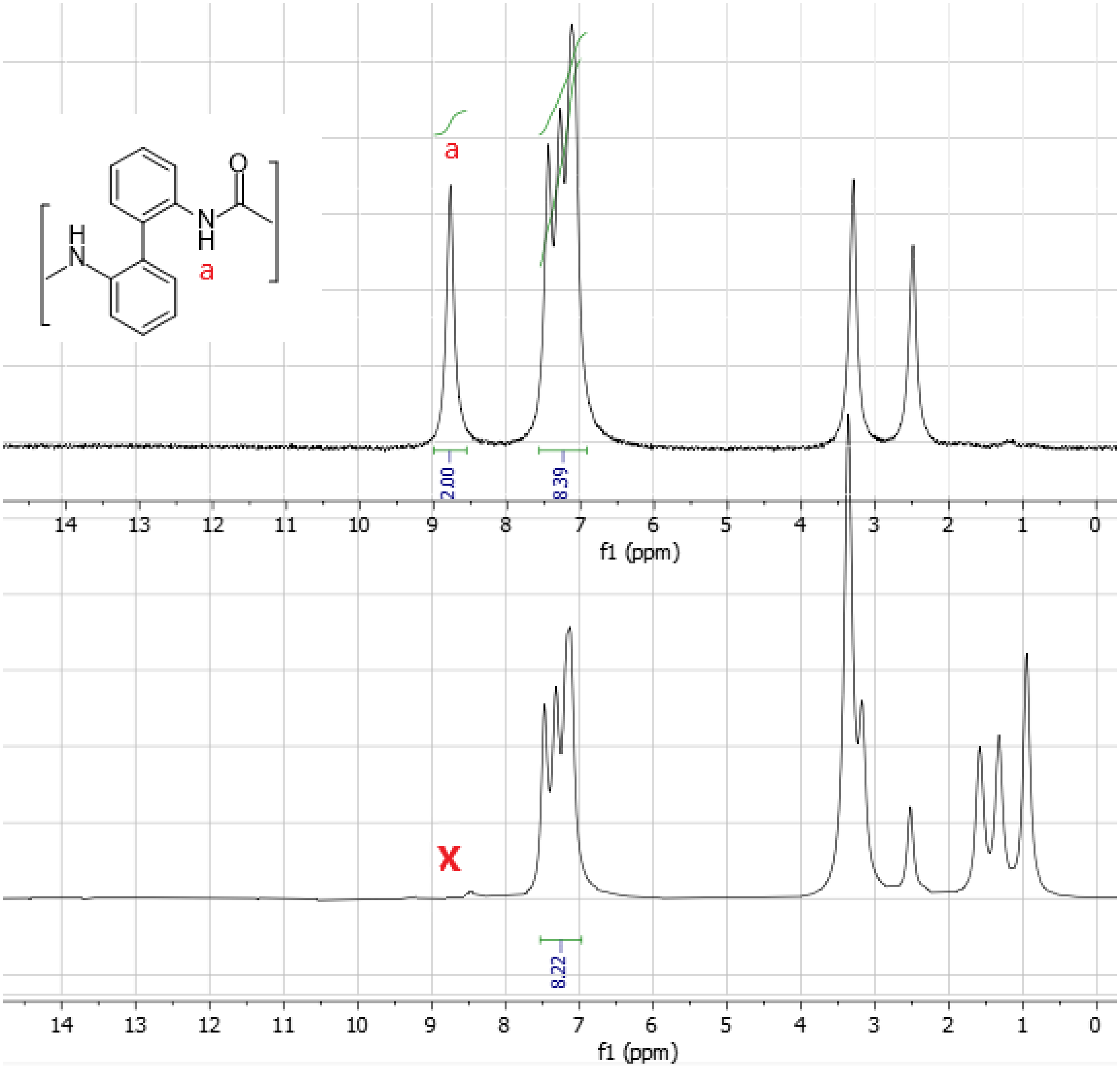

Figure S11. ^{1}**H NMR spectrum of the (4) in DMSO-d_6 at r.t. with additional TBAF**

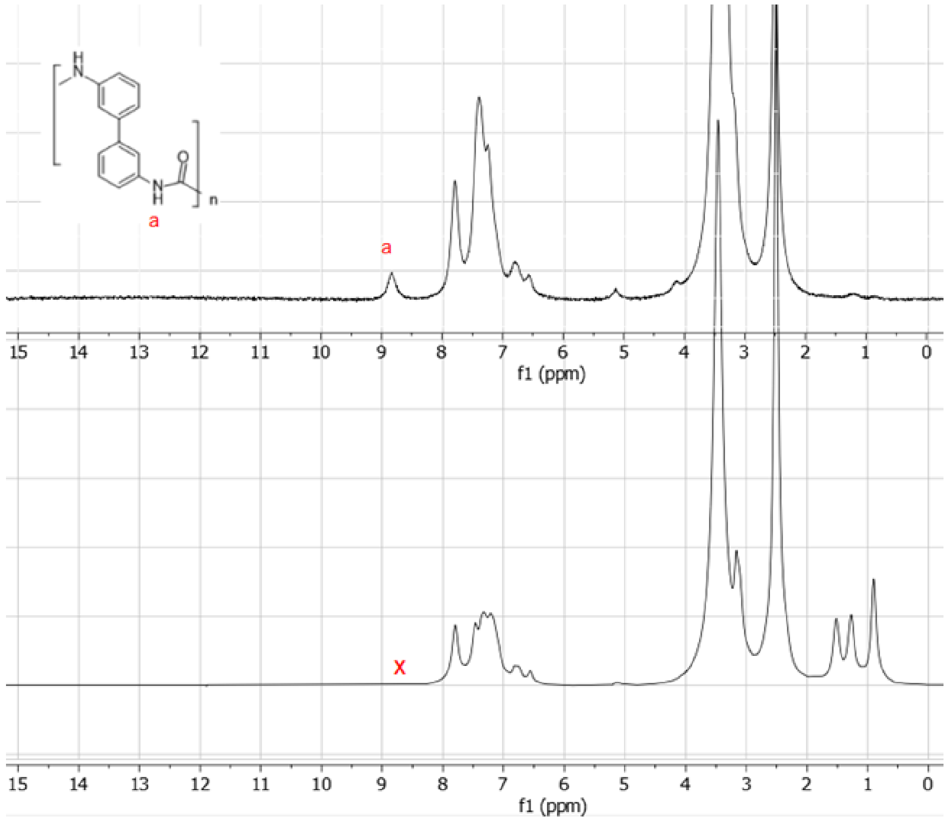

Figure S12. ^{1}H NMR spectrum of the (5) in DMSO-d_6 at r.t. with additional TBAOH

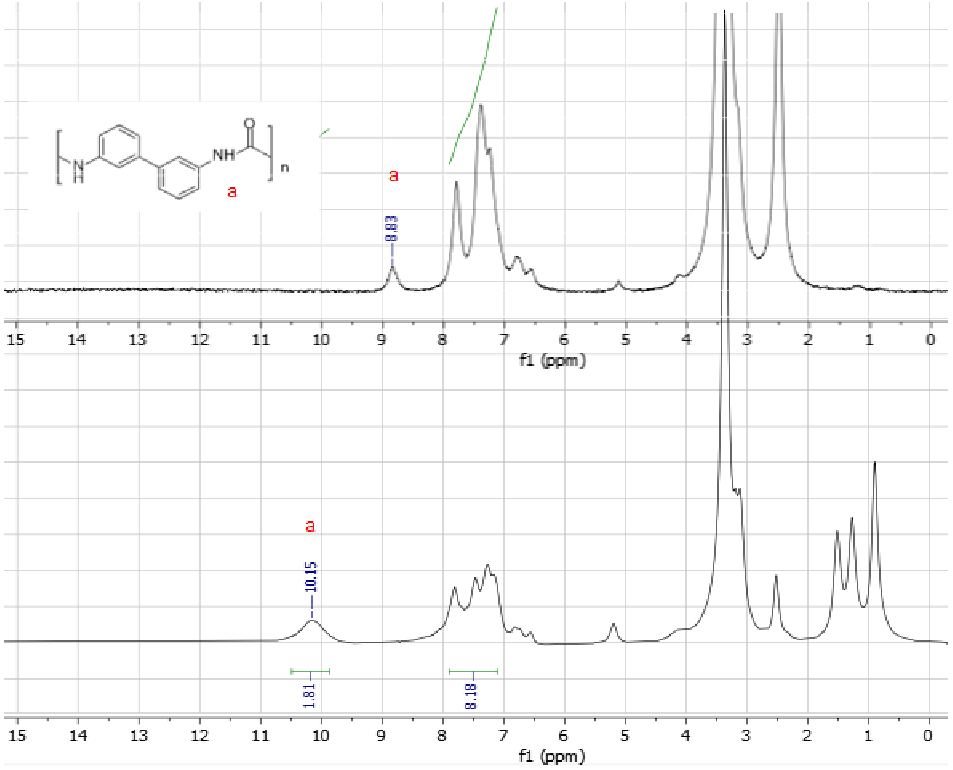

Figure S13. ^{1}H NMR spectrum of the (5) in DMSO-d_6 at r.t. with additional TBAF

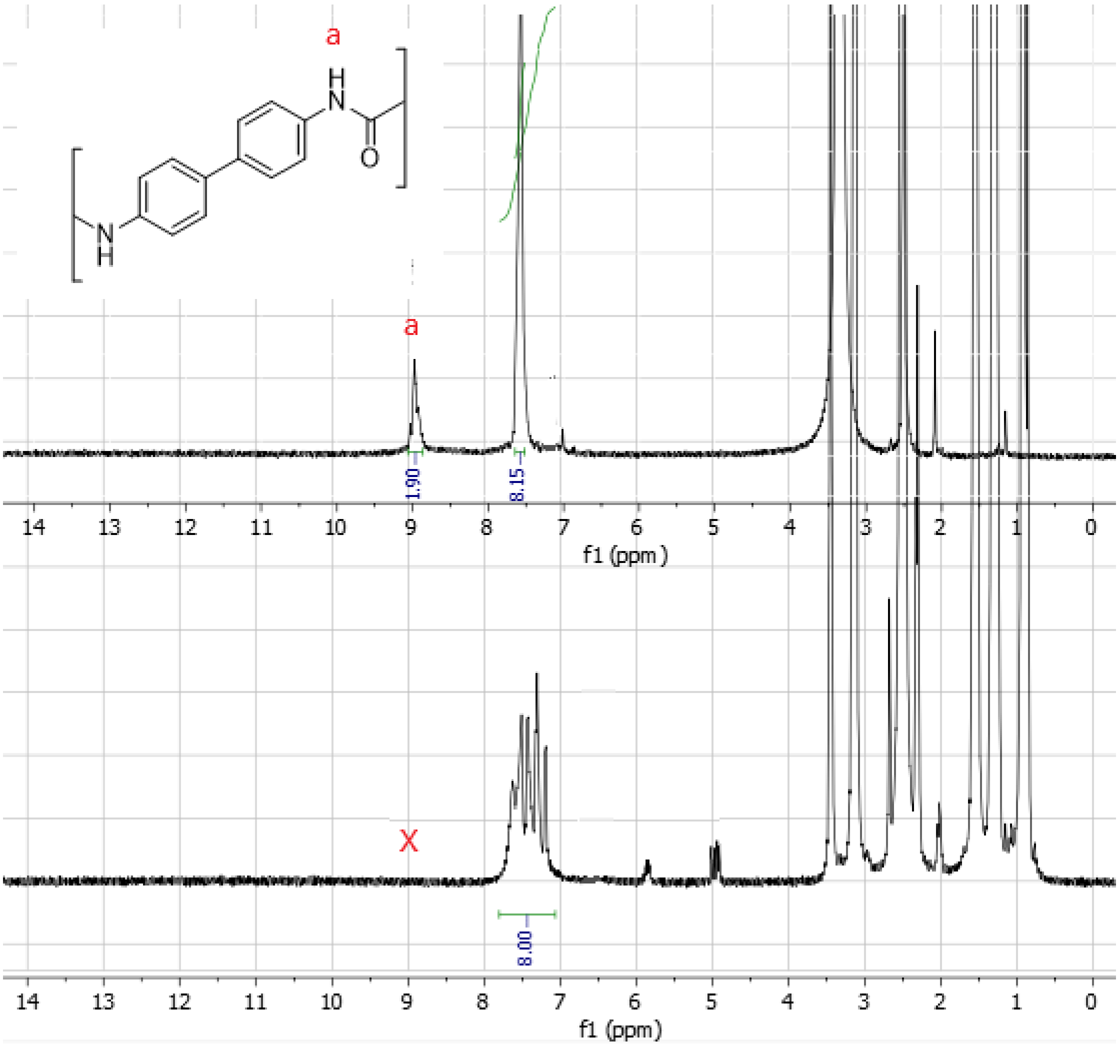

Figure S14. **^{1}H NMR spectrum of the (9) in DMSO-d_6 at r.t. with additional TBAOH**

Table S1 Photophysical properties of the PUs 4, 5, 6 and their complexes with F (-)

Polymer/complex		Absorption maxima, nm	Emission maxima, nm
**4**	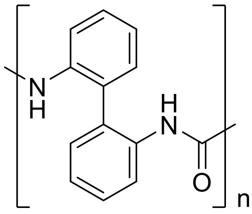	297	430
**4+F(-)**		340 (sh)	432, 551
**5**		299 (sh)	352, 406

**5+F(-)**	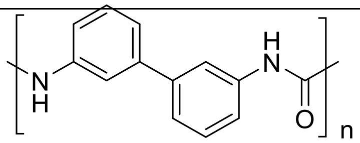	319	508
**6**	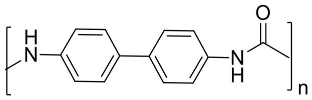	327 (pl)	365 (pl)
**6+F(-)**		340 (sh)	385

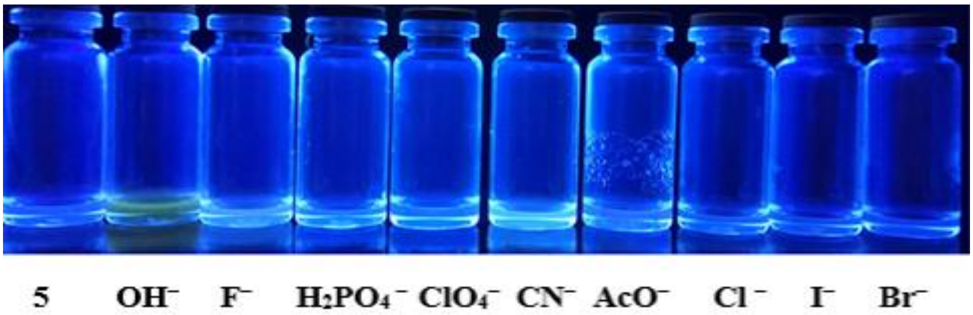

Figure S15. Fluorescence response of PU 4

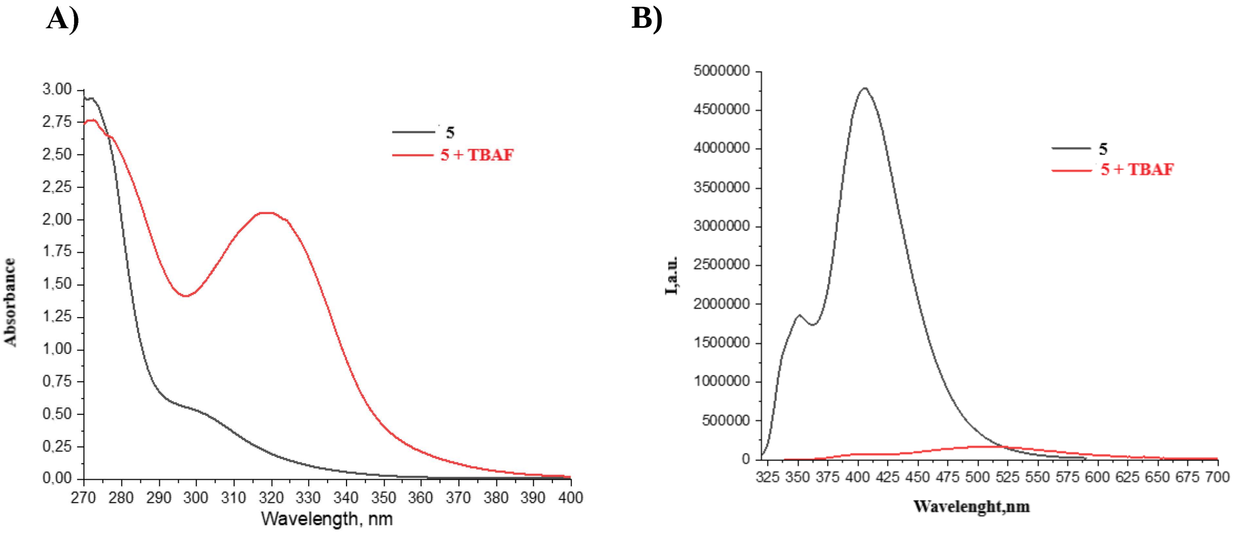

Figure S16. Change in absorption (A) and emission (B) spectra of PU 5 in DMSO in the presence of TBAF.

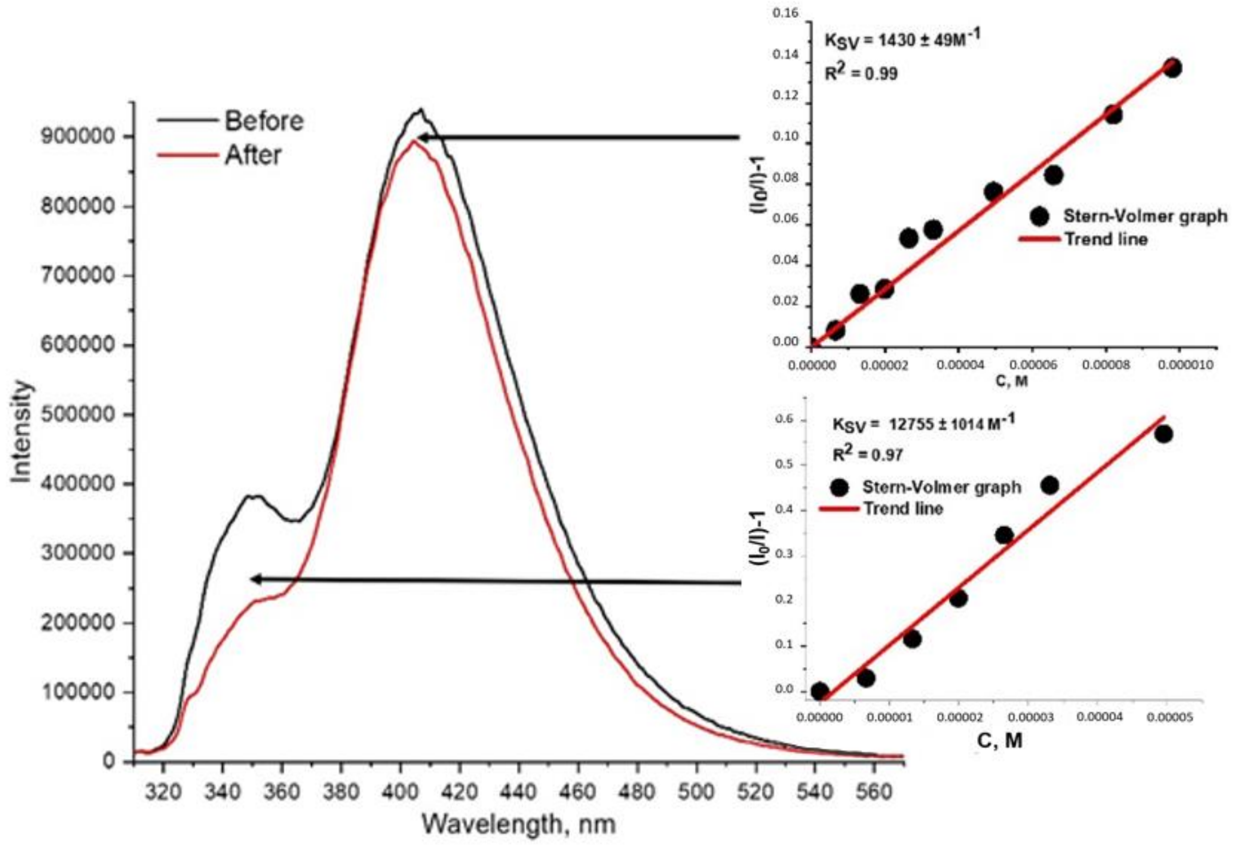

Figure S17. Fluorescence quenching effect of TBAF on the PU (5).

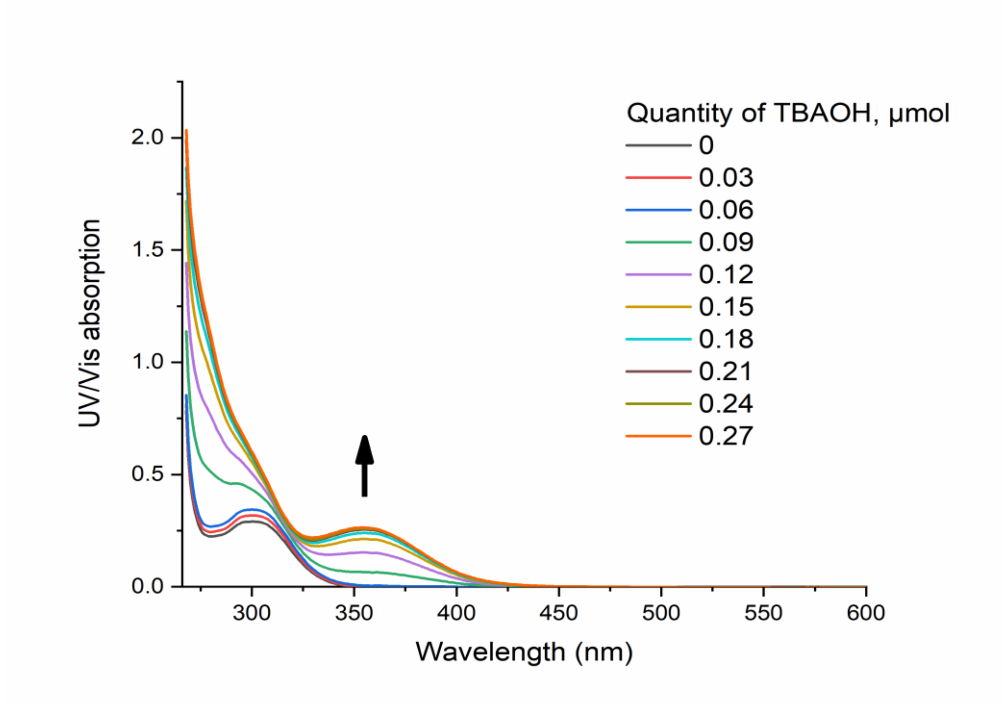

Figure S18. Change in absorption of PU (4) in DMSO in the presence of TBAOH.

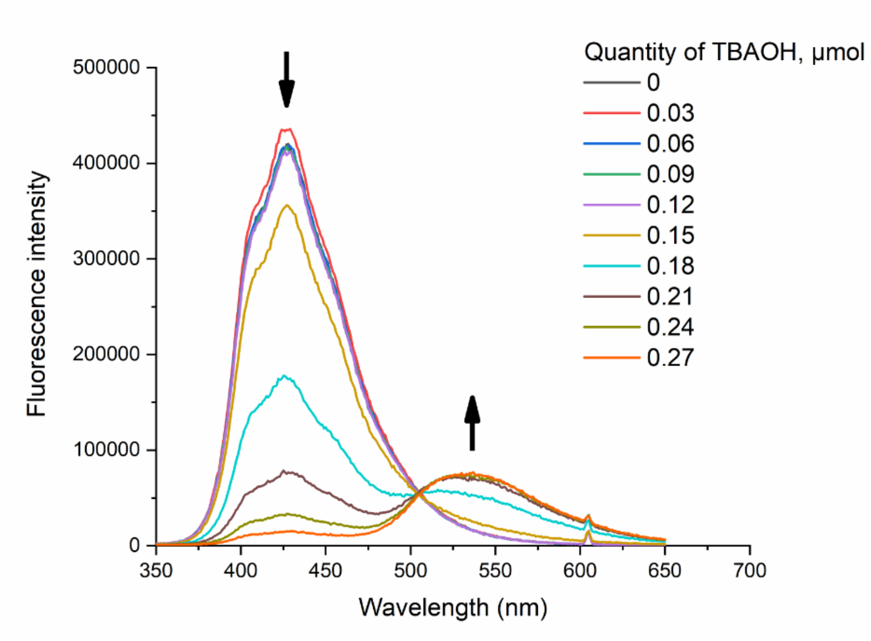

Figure S19. Change in emission PU (4) in DMSO in the presence of TBAOH.

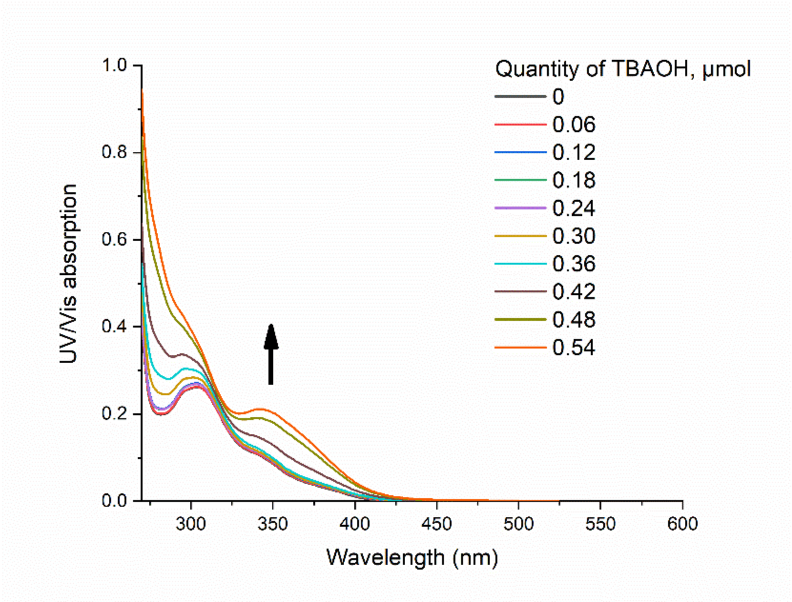

Figure S20. Change in absorption of PU (7) in DMSO in the presence of TBAOH.

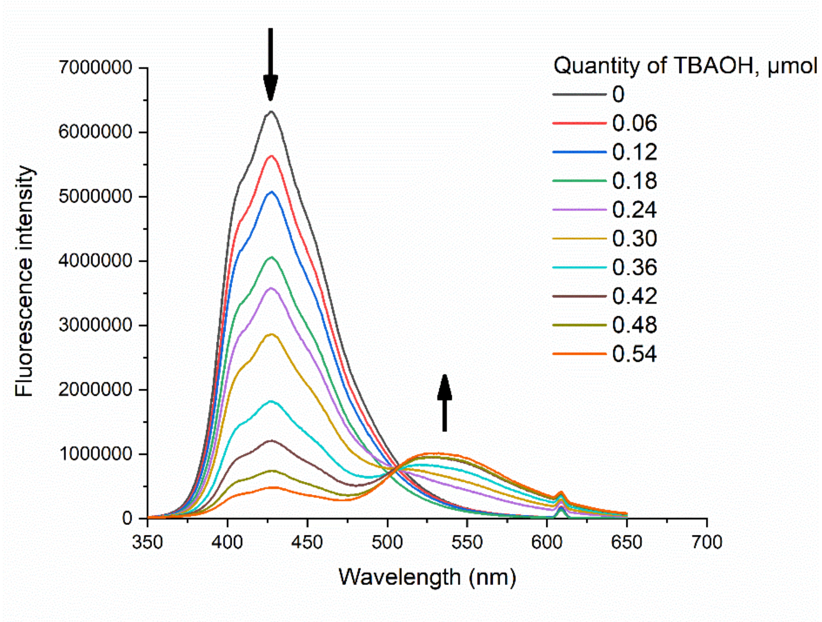

Figure S21. Change in emission PU (7) in DMSO in the presence of TBAOH.

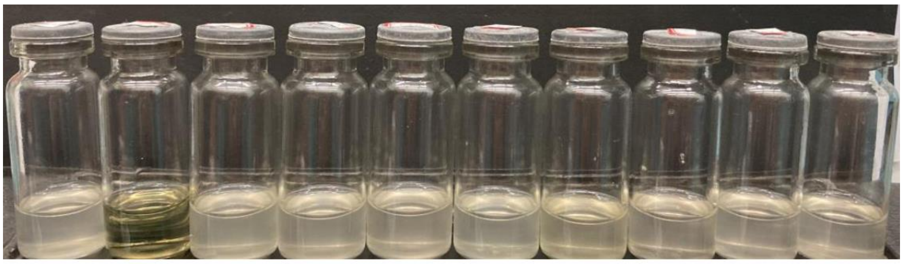

Free OH$^-$ F$^-$ $H_2PO_4^-$ ClO_4^- CN$^-$ AcO$^-$ Cl$^-$ I$^-$ Br$^-$

Figure S22. Colorimetric response of PU 6 towards anions in DMSO solution under Daylight.

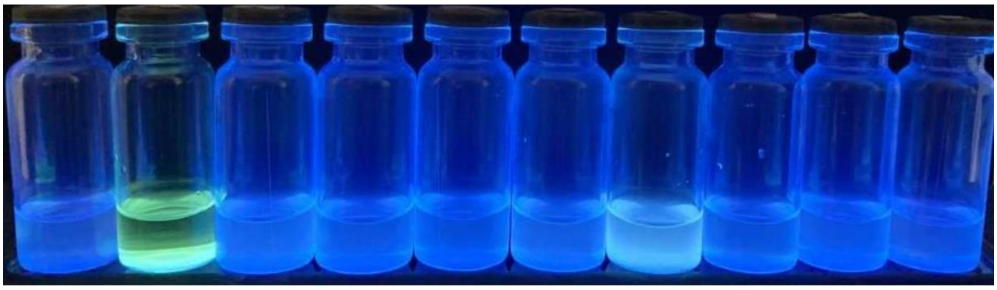

Free OH$^-$ F$^-$ $H_2PO_4^-$ ClO_4^- CN$^-$ AcO$^-$ Cl$^-$ I$^-$ Br$^-$

Figure S23. Fluorescence response of PU 6 toward anions in DMSO solution under UV (λex = 365 nm).

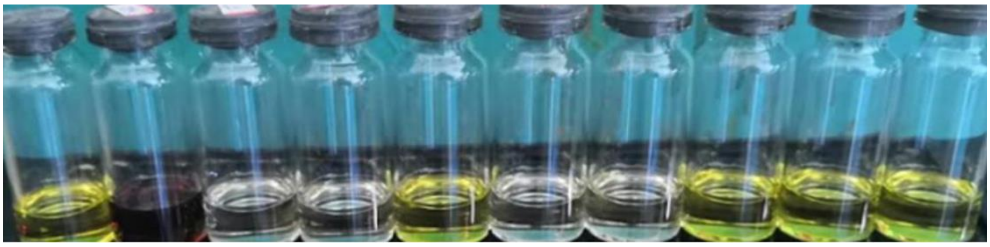

Figure S24. Colorimetric response of PU 8 towards anions in DMSO solution under Daylight

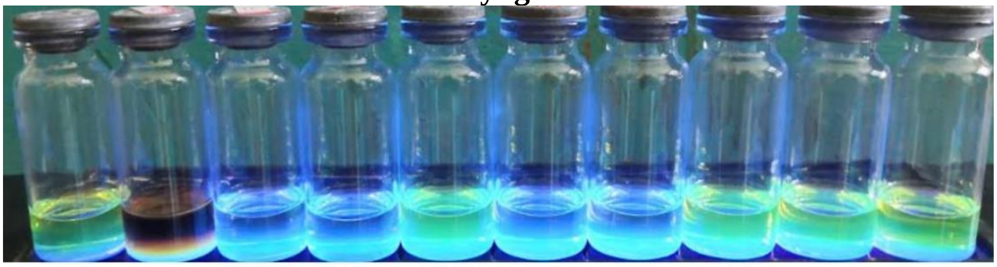

8 OH^- F^- $H_2PO_4^-$ ClO_4^- CN^- AcO^- Cl^- I^- Br^-

Figure S 25. Fluorescence response of PU 8 toward anions in DMSO solution under UV (λex = 365 nm).

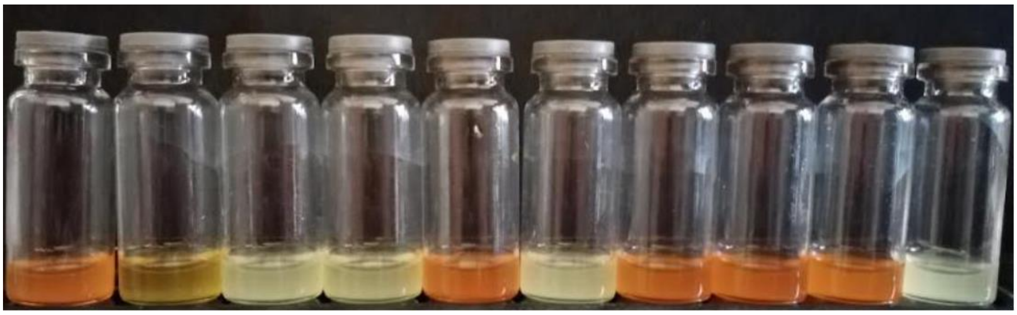

Figure S26. Colorimetric response of PU 9 towards anions in DMSO solution under

Daylight

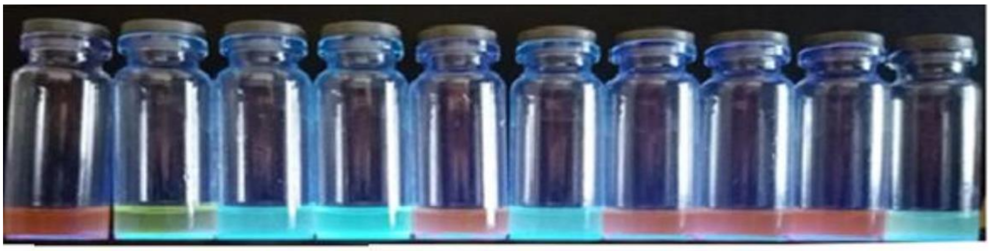

9 OH^- F^- $H_2PO_4^-$ ClO_4^- CN^- Cl^- I^- Br^- AcO^-

Figure S27. Fluorescence response of PU 9 toward anions in DMSO solution under UV (λex

= 365 nm)

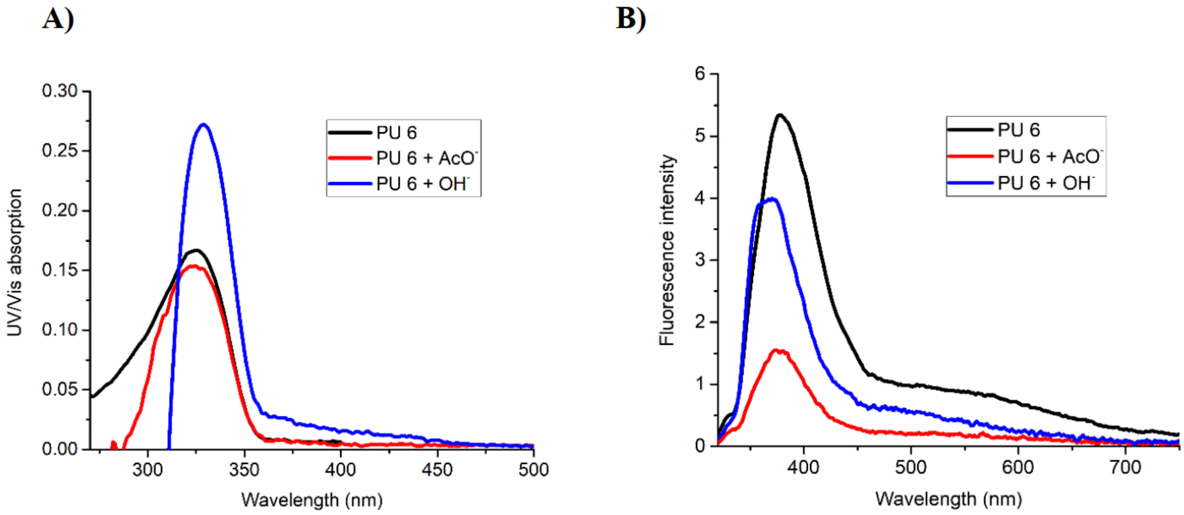

Figure S28. Change in absorption (A) and emission (B) spectra of PU 6 in DMSO in the presence of various anions.

General method for the synthesis of Polyurea's 7-9.

Isomeric 2,2(1)-, 3,3(2)- or 4,4 (3) -diaminobiphenyls (1 eq.), triphosgene (1.5 eq), 4-N,Ndimethylaminobenzaldehyde (0.1 eq) and K_2CO_3 (7 eq.) were reacted in 25 ml stainless still milling jar with 4 balls 10 mm stainless still milling balls for 4 hours at a speed rotation of 500 rpm. After that the reaction mixture was washed by 10% aqueous solution of HCl, water, EtOH and acetone.

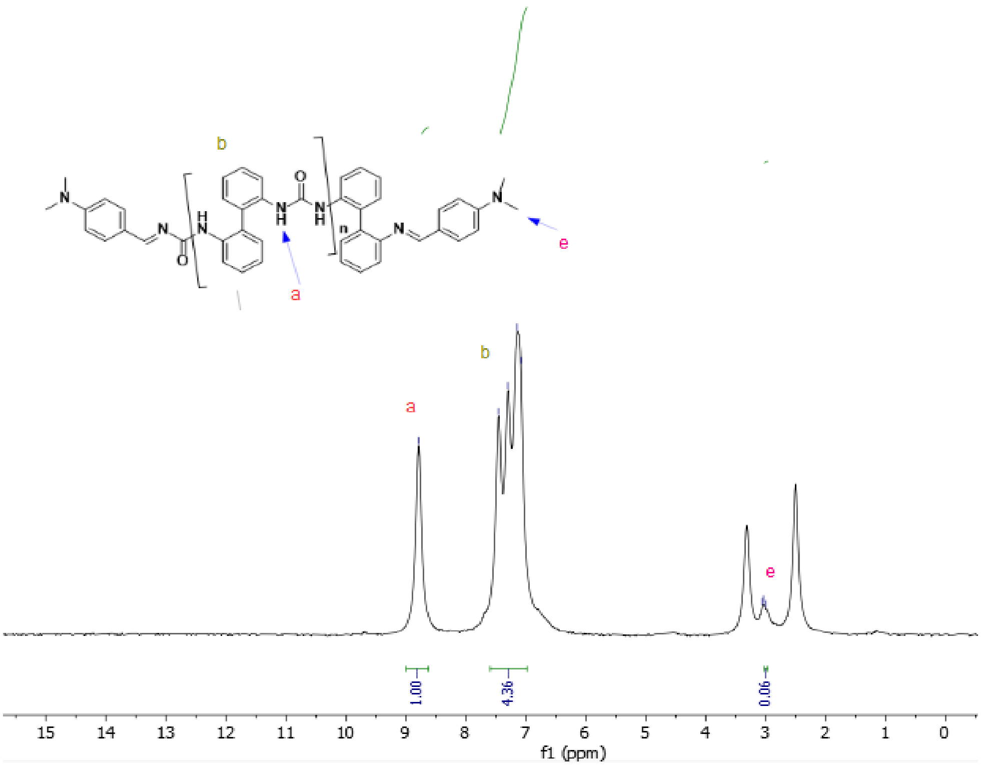

Figure S29. ^{1}H NMR of PU 7 in DMSO-d_6

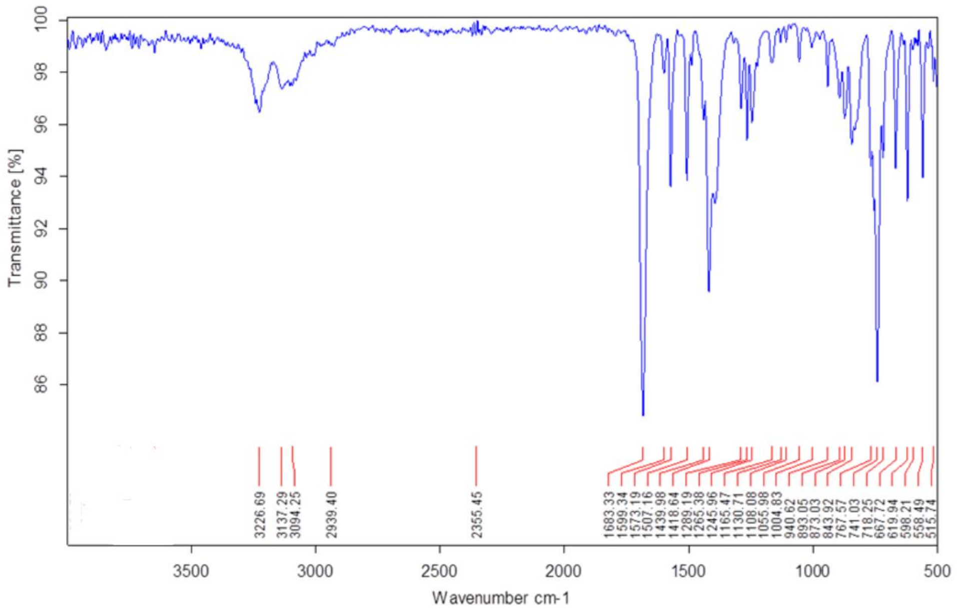

Figure S30. IR-spectra of PU 7

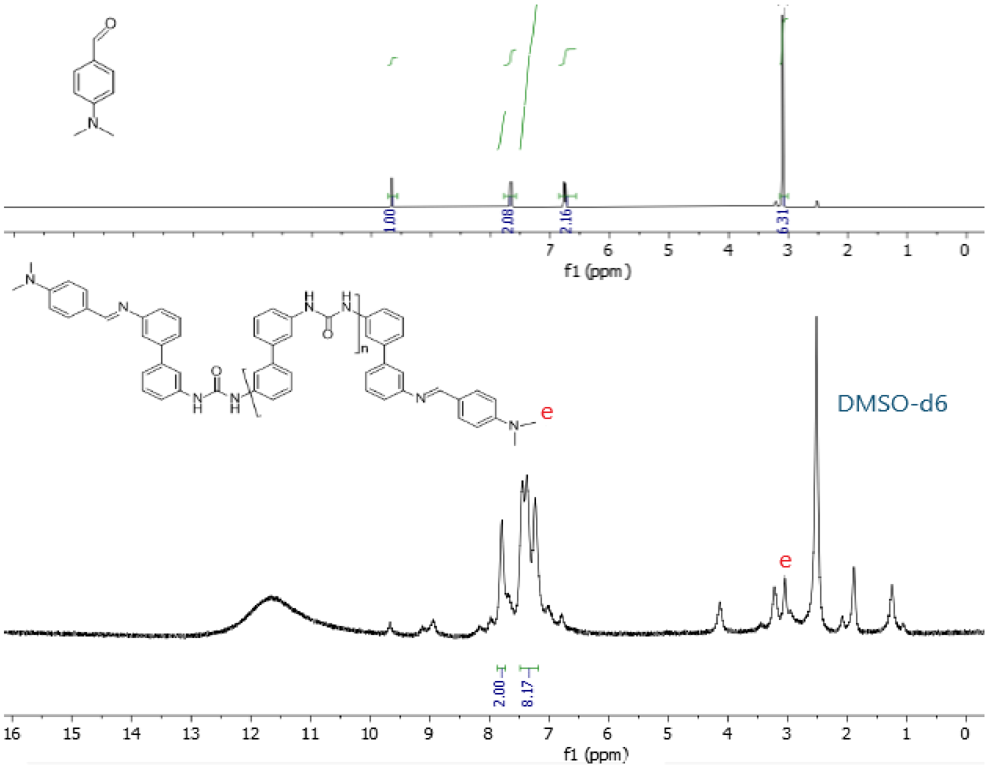

Figure S31. ^{1}H NMR spectra of PU 8 in DMSO-d_6

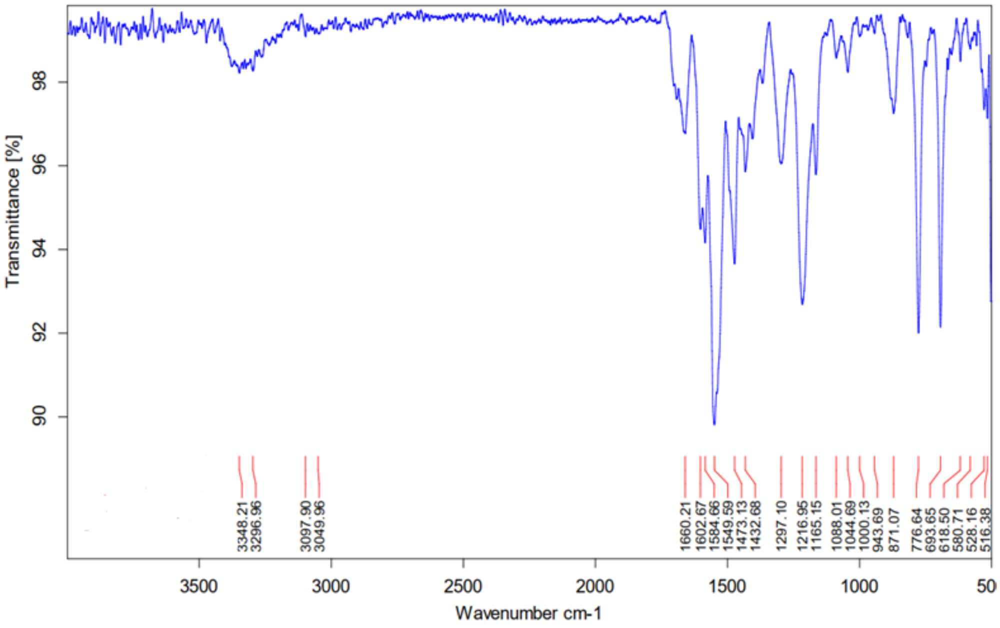

Figure S32. IR spectra of PU 8

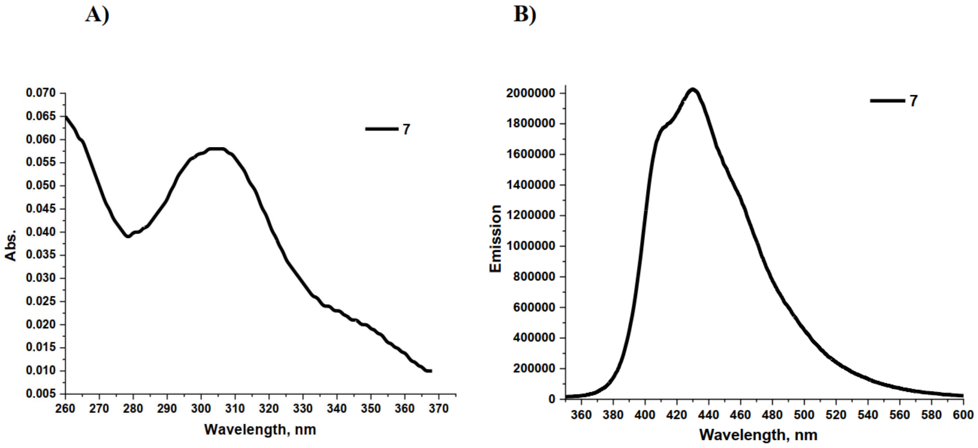

Figure S33:Absorption (A) and emission (B) spectra of PU 7 in DMSO

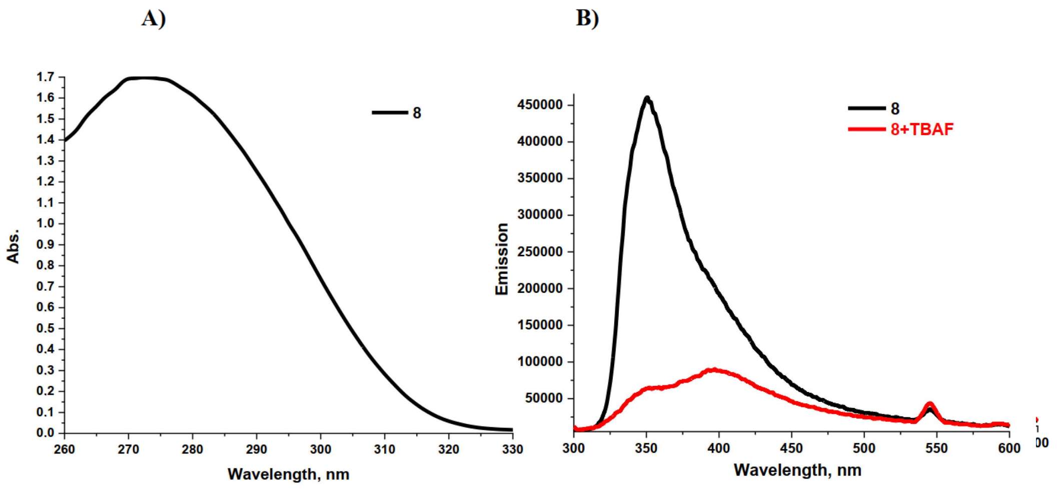

Figure S34. Absorption spectra (A) and change in emission spectra (B) of PU 8 in DMSO in the presence of TBAF

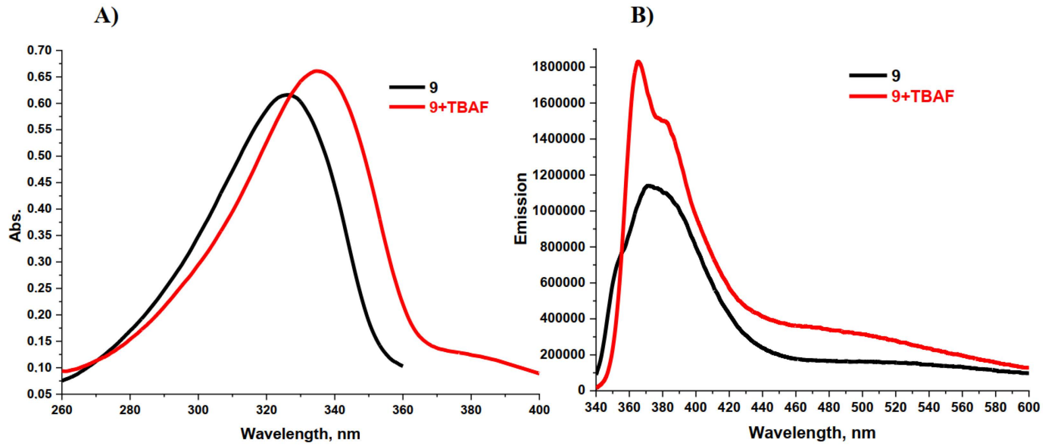

Figure S35. Change in absorption (A) and emission (B) spectra of PU 9 in DMSO in the presence of TBAF

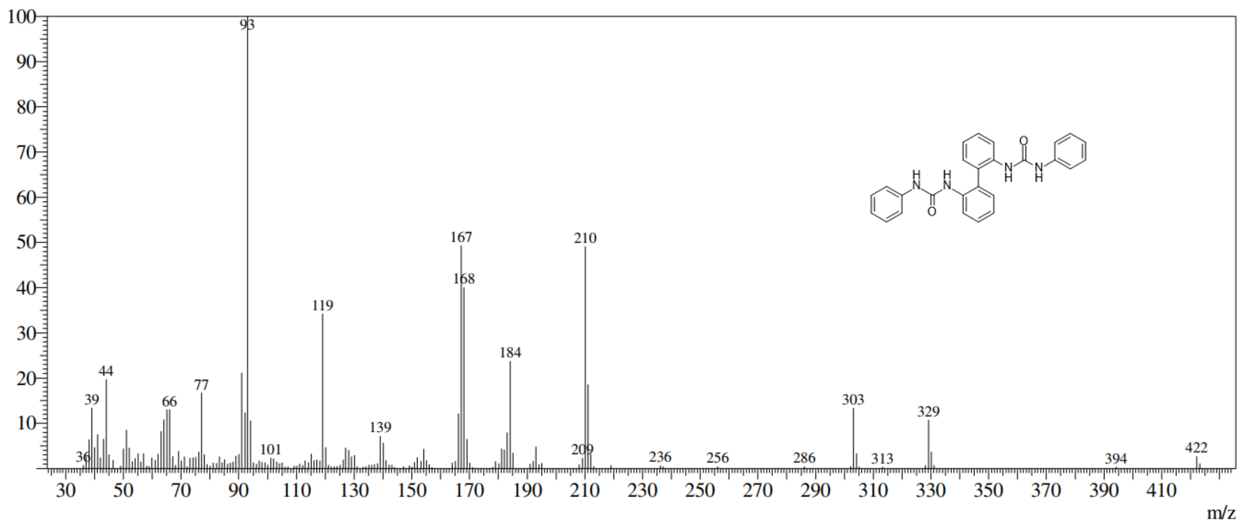

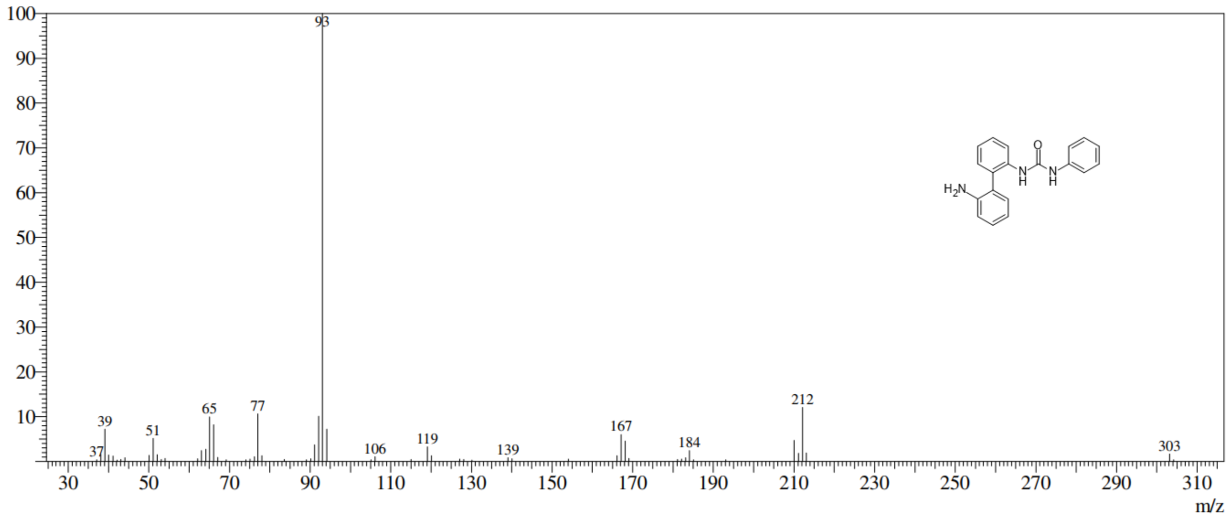

Figure S36. mass spectra of 1,1'-([1,1'-biphenyl]-2,2'-diyl)bis(3-phenylurea) and1-(2'-amino-[1,1'-biphenyl]-2-yl)-3-phenylurea.

Table S2. Results of intraday and interday experiments on synthesis of PUs

	PU4	PU5	PU6
Intraday			
Mean ± SD	75.67 ± 1.2472	68.67 ± 0.9428	93.00 ± 1.6330
RSD	2.02	1.68	2.15
Interday			
Mean ± SD	76.00 ± 0.8165	68.33 ± 1.6997	93.67 ± 1.2472
RSD	1.32	2.22	1.63
p-value			
p-value	0.42	0.29	0.85

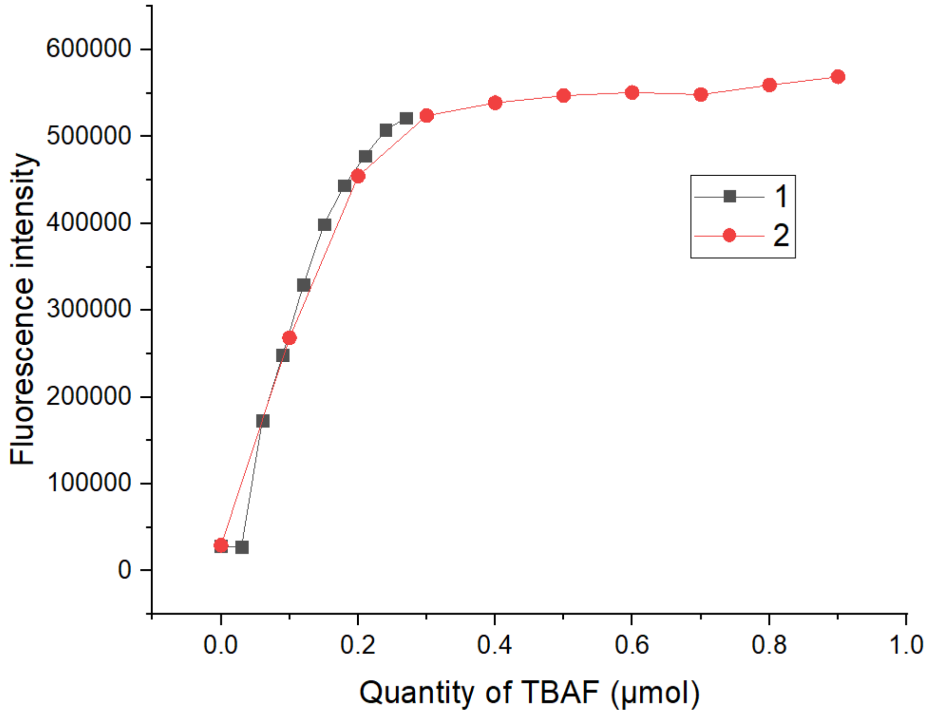

Figure S37. Change of FL emission intensity of PU **4** at 530 nm with different aliquot of TBAF Table S3. Data for Figure S37.

# 1		# 2	
Quantity of TBAF in µmol	FL Intensity	Quantity of TBAF in µmol	FL Intensity
0	28250	0	29456
0.03	27826	0.1	268320
0.06	172610	0.2	454730
0.09	248880	0.3	524140
0.12	329080	0.4	539040
0.15	399070	0.5	547310
0.18	444050	0.6	551000
0.21	477810	0.7	548640
0.24	507790	0.8	559430
0.27	521710	0.9	568290

Computational details

The quantum chemical calculations including full geometry optimization of all model structures were carried out at the ωB97XD/CEP-121G level of theory with the help of the Gaussian-09 program package [1]. No symmetry restrictions were applied during the geometry optimization procedure. The Hessian matrices were calculated analytically for all optimized model structures to prove the location of correct minima on the potential energy surface (no imaginary frequencies were found in all cases).

Table S4. Calculated Gibbs free energies for optimized equilibrium model structures

Model structure	Gibbs free energy, a.u.
**ACO^-**	-45.022612
**Br^-**	-13.481468
**Cl^-**	-15.004344
**ClO_4^-**	-78.305417
**CN^-**	-15.528862

**F^-**	-24.170916
**$H_2PO_4^-$**	-71.560313
**I^-**	-11.559448
**OH^-**	-16.504252
**PU4···ACO^-**	-161.269585
**PU4···Br^-**	-129.712888
**PU4···Cl^-**	-131.244522
**PU4···ClO_4^-**	-194.521853
**PU4···CN^-**	-131.770029
**PU4···F^-**	-140.461544
**PU4···$H_2PO_4^-$**	-187.795341
**PU4···I^-**	-127.783640
**PU4**	-116.200781
**PU4···OH^-**	-132.801763
**PU5···ACO^-**	-161.278974
**PU5···Br^-**	-129.720983
**PU5···Cl^-**	-131.252858
**PU5···ClO_4^-**	-194.526387
**PU5···CN^-**	-131.778292
**PU5···F^-**	-140.467525
**PU5···$H_2PO_4^-$**	-187.799701
**PU5···I^-**	-127.792294
**PU5**	-116.199835
**PU5···OH^-**	-132.810579
**PU6···ACO^-**	-161.280317
**PU6···Br^-**	-129.721682
**PU6···Cl^-**	-131.253835
**PU6···ClO_4^-**	-194.528556
**PU6···CN^-**	-131.780227
**PU6···F^-**	-140.468666
**PU6···$H_2PO_4^-$**	-187.804323
**PU6···I^-**	-127.792007
**PU6**	-116.200136
**PU6···OH^-**	-132.811808

References.

1. Frisch, M.J.; Trucks, G.W.; Schlegel, H.B.; Scuseria, G.E.; Robb, M.A.; Cheeseman, J.R.; Scalmani, G.; Barone, V.; Petersson, G.A.; Nakatsuji, H.; et al. Gaussian 09, Revision C.01, Gaussian, Inc., Wallingford, CT, 2010.
